# A High Throughput and User-Friendly Computational Tool for Quantifying and Visualizing Symmetry Variation in Image Datasets

**DOI:** 10.1007/s11538-026-01754-9

**Published:** 2026-10-07

**Authors:** Liam D. O’Brien, Punit Gandhi, Maria-Veronica Ciocanel, Adriana T. Dawes

**Affiliations:** 1 Department of Mathematics, The Ohio State University, Columbus, OH USA; 2 Department of Mathematics and Statistics, Virginia Commonwealth University, Richmond, VA USA; 3 Department of Mathematics, Duke University, Durham, NC USA; 4 Department of Biology, Duke University, Durham, NC USA; 5 Department of Molecular Genetics, The Ohio State University, Columbus, OH USA

**Keywords:** Transformation information, Asymmetry quantification, Approximate symmetry, Symmetry classification, Image analysis, Floral symmetry

## Abstract

Symmetry is a fundamental property of biological systems, yet it is commonly reduced to discrete, subjective classifications that fail to capture the continuous and graded nature of symmetry in real organisms. We present a high-throughput, user-friendly computational framework for quantifying and visualizing biological symmetry as a continuous variable using *transformation information* ($\mathcal{T}\mathcal{I}$), an information theoretic measure that quantifies the divergence between an object and its transformed self. We establish key mathematical properties of $\mathcal{T}\mathcal{I}$, including invariance, equivariance, and Lipschitz continuity, that guarantee robustness to transformations such as translation, rotation, reflection, and rescaling, enabling reliable symmetry quantification across heterogeneous image datasets without manual landmarking or image alignment. Building on this theoretical foundation, we introduce a computational pipeline that automates symmetry center detection, $\mathcal{T}\mathcal{I}$ curve computation, and visualization of the symmetry data using functional principal component analysis (fPCA). We demonstrate the utility of this framework on two biological datasets: a large collection of angiosperm flower images, where we investigate rotational and reflectional symmetries, and a dataset of simulated fern images, where we investigate fractal self-similarity under scaling and translation. Our analysis of the floral dataset provides novel evidence of convergent evolution. In both datasets, fPCA of the resulting $\mathcal{T}\mathcal{I}$ profiles reveals that symmetry is best characterized as a continuum rather than a set of discrete classes, and there is subtle morphological variation that is not captured by traditional categorical approaches. Together, these results suggest that $\mathcal{T}\mathcal{I}$ provides a mathematically principled and biologically informative foundation for integrating symmetry into quantitative evolutionary, ecological, and developmental models.

**Supplementary Information:**

The online version contains supplementary material available at https://doi.org/10.1007/s11538-026-01754-9.

## Introduction

Symmetry is a fundamental property of living systems, manifesting across multiple temporal and spatial scales, and driving biological functions such as fecundity and evolvability. In flowering plants, for example, symmetries in floral structures are tightly linked to their reproductive behavior and ecological interactions (Endress 1999; Luo et al. 1996; Neal et al. 1998; Sargent 1539; Fenster et al. 2004; Nikkeshi et al. 2015; Wang et al. 2023).

Uncovering the role of symmetry in biology relies on large-scale phenotyping; however, researchers often default to treating symmetry as a discrete, system-specific variable and classify specimens based on subjective visual criteria. Flowers, for instance, are typically grouped as actinomorphic (radial symmetry) or zygomorphic (bilateral symmetry); yet, many flowers fit neither category, making the classification inadequate. Such categorical approaches bias symmetry measurements, prevent meaningful comparison across heterogeneous populations, and impose an artificial structure on organisms that are inherently variable. By reducing a rich spectrum of symmetry to a set of subjective categorical labels, scientists risk discarding subtle information that may hold the key to understanding how symmetry affects biological fitness.

Mathematical descriptions of symmetry have traditionally relied on the rigorous framework of group theory, which defines symmetry as invariance under a specified transformation. While this formalism is powerful, its application to biological data is severely limited. Group theory classifies objects by the subgroup of transformations they are invariant under, partitioning them into discrete symmetry types such as $C_n$ (cyclic) or $D_n$ (dihedral). Since biological symmetries are rarely exact, most natural objects would not belong to any symmetry class under this classification. For example, a flower with nearly but not perfect five-fold rotational symmetry is assigned to the same class as a completely asymmetric object. Moreover, even among objects sharing a symmetry class, meaningful morphological differences are discarded. As a result, rigid group-theoretic definitions fail to capture the graded and continuous nature of symmetry in real biological data.

Methods for measuring approximate symmetry and morphology in biology have relied heavily on landmark-based morphometrics or outline analyses (Klingenberg 2002), which quantify deviations from ideal symmetry by comparing homologous points or structures reflected across some axis. More recent work has aimed to automate and generalize symmetry measurements. For instance, Savriama and Klingenberg (2011) extended landmark-based morphometrics to consider general symmetries. These established methods still require expert input to select biologically-meaningful landmarks and often assume bilateral symmetry across a user-selected axis. To address these issues, Gandhi et al. (2021) proposed using an information-theoretic measure called *transformation information* ($\mathcal{T}\mathcal{I}$) (Vstovsky 1997) for biological data. $\mathcal{T}\mathcal{I}$ quantifies symmetry by comparing an object to its transformed self across a broad set of transformations. Rather than assigning each specimen to a discrete symmetry category, it represents symmetry with a function that varies continuously (in function space) and captures the full symmetry profile. $\mathcal{T}\mathcal{I}$ does not require segmentation of the object or selection of key landmarks, avoiding two steps that are often difficult or subjective for biological imagery. However, this measure still requires that a meaningful set of symmetry transformations is selected, that the object of interest is isolated against a uniform black background, and that the center of the object is selected. This approach trades difficult or subjective steps for more tractable and unbiased ones, which can often be automated. Bermingham et al. (2023) also recently proposed the use of the *extended persistent homology transform* to automatically quantify approximate symmetries. This approach is well-suited for capturing topological changes in binary images or shapes under a symmetry transformation, whereas the $\mathcal{T}\mathcal{I}$ approach captures changes in scalar-valued functions under a symmetry transformation (e.g. pixel intensity for an image).

Ciocanel et al. (2025) extended the $\mathcal{T}\mathcal{I}$ framework by studying transitions in symmetry, proposing a formal analogy with bifurcation theory. This study used $\mathcal{T}\mathcal{I}$ to characterize critical changes in symmetry across model-generated developmental patterns and demonstrated the sensitivity of $\mathcal{T}\mathcal{I}$ to subtle changes in morphology that are not easily detected by eye.

In this work, we advance the $\mathcal{T}\mathcal{I}$ paradigm by developing a robust, high-throughput, and user-friendly pipeline for symmetry analysis of image data. We first establish mathematically-desirable properties of $\mathcal{T}\mathcal{I}$, ensuring that the method works for diverse data types with minimal preprocessing (Sect. 3). Building on this theoretical foundation, we present a general computational framework and accompanying software for measuring and comparing the symmetries of image data (GitHub 2025) (Sect. 4).

We then illustrate the versatility of the approach by applying our computational framework to two distinct systems: a large dataset of angiosperm flower images and a generated dataset of ferns (Sect. 5). In the floral dataset, our continuous, function-valued symmetry measure reveals something biologically meaningful: across flowering plants, distantly-related clades contain flowers that span the gamut of symmetry types, suggesting floral symmetries evolved independently across lineages and therefore carry real functional significance, likely tied to survival in specific ecological niches. The application to simulated ferns demonstrates the flexibility of our method, which can capture symmetry under arbitrary transformations. We investigate the fractal symmetry of ferns and show that fern symmetry also exists along a continuum. Together, these applications illustrate that our framework can detect subtle morphological features that may not be observable using traditional discrete classifications, allowing researchers to move towards developing more sophisticated statistical models that can correlate a continuous, function-valued symmetry measure to functional properties in organisms.

## Introduction to Transformation Information ($\mathbf {\mathcal{T}\mathcal{I}}$)

Approximate symmetry can be measured by comparing an object to a transformed version of the object. When the transformed object is similar to the original, the object is approximately symmetric under that transformation. Objects of interest in biology may include concentrations of biochemicals, patterns of cell fates, or image datasets, which can all be represented with a distribution μ(x) that takes a spatial coordinate *x* as input and outputs a real number. Furthermore, we can transform μ(x) by composing it with an appropriate (invertible) transformation $T_a$. A distribution μ(x) transformed by $T_a$ is given by the distribution $\mu (T_a^{-1}x)$, which we denote $T_a \mu$ (as in representation theory, where $T_a$ can be thought of as a acting on μ (Golubitsky et al. (2012), Ch. 7). Transformation information (defined below) measures the divergence between μ and $T_a \mu$, yielding a value of zero if they are identical and a large value if they are very different. Thus, the value of $\mathcal{T}\mathcal{I}$ provides a measure of deviation of the object from being (exactly) symmetric.

### Definition 1

*(Transformation information)* Let $\mu : D \rightarrow \mathbb {R}_{>0}$ with $D\subset \mathbb {R}^n$ be a measurable and positive-valued function, and let $\{T_a: \mathbb {R}^n \rightarrow \mathbb {R}^n\}_{a\in \mathcal {A}}$ be a set of invertible transformations smoothly parameterized in *a*. Then the transformation information of μ under the family of transformations $\{T_a\}$ is given by

1 $$\begin{aligned} \mathcal{T}\mathcal{I}[\mu ,T_a] =\int _{\widetilde{D}} \mu (x)\log \left( \frac{\mu (x)}{T_a\mu (x)}\right) \ dx \,, \end{aligned}$$

where $$\widetilde{D}=D\cap T_a(D)$$.

The measure is analogous to quantifying the relative entropy between μ and $T_a\mu$, which can be interpreted as the inefficiency in describing the system with the distribution $T_a\mu$ when the true distribution is μ. Relative entropy provides a conceptually-rich framework that connects symmetry with ideas from statistical mechanics and dynamical systems. Alternative definitions of $\mathcal{T}\mathcal{I}$, which require μ to be normalized or alter the region of integration, have been considered and are discussed in Sect. A.1.

### Remark 1

The assumption that μ is measurable guarantees that the integral is defined. In applications to data, this assumption is typically satisfied.

### Remark 2

When data consists of images, a continuous signal is sampled on a discrete domain *D*. When *D* is discrete, $\widetilde{D}$ is empty for many transformations $T_a$, so $\mathcal{T}\mathcal{I}[\mu ,T_a]$ can vanish even for highly asymmetric data. We address this issue in Sect. 4.3.

### Remark 3

To ensure that $\mathcal{T}\mathcal{I}[\mu , T_a] <\infty$, we often require that *D* is compact (equivalently, closed and bounded) or that μ is bounded below by some positive value.

### Remark 4

Although we primarily observe rotational and bilateral symmetry in biological systems, transformation information can measure the symmetry of an object under any invertible transformation. We often assume that $\{T_a\}$ is a subset of the invertible affine transformations on $\mathbb {R}^n$, denoted $\text {Aff}(n)$; however, we can certainly consider more general transformations such as the set of diffeomorphisms from $\mathbb {R}^n \rightarrow \mathbb {R}^n$.

### Remark 5

An affine transformation on $\mathbb {R}^n$

$$\begin{aligned} T_a(x)=Ax+b \end{aligned}$$

can be written in *homogeneous coordinates* with the (n+1)×(n+1) augmented matrix below.

$$\begin{aligned} \begin{pmatrix} A & b \\ 0^T & 1 \end{pmatrix} \begin{pmatrix} x \\ 1 \end{pmatrix} = \begin{pmatrix} T_a(x)\\ 1 \end{pmatrix} \end{aligned}$$

In this notation, composition of affine transformations is equivalent to matrix multiplication. Furthermore, the determinant of an affine transformation is defined as the matrix determinant in homogeneous coordinates.

### Definition 2

*(Local Minima and Maxima)* Let $f: X \rightarrow \mathbb {R}$ be a function on a topological space *X*. x∈X is a local minimum (respectively, maximum) if there exists some neighborhood of *x*, $\mathcal {N}_x$ such that for every $y\in \mathcal {N}_x$, f(x)≤f(y) (respectively, f(x)≥f(y)).

### Definition 3

**Fig. 1:**
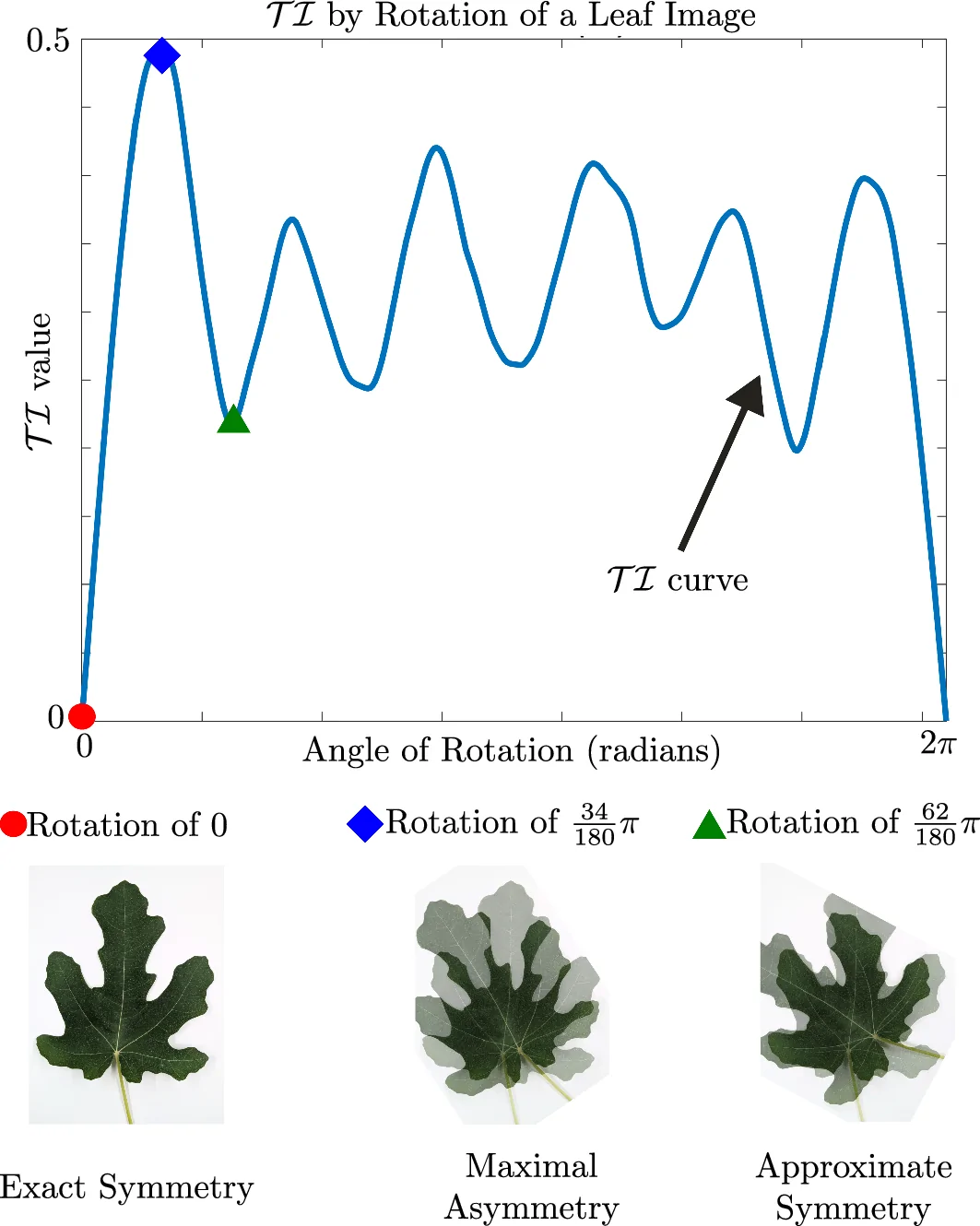
We represent the leaf image by a positive-valued function μ and compute $\mathcal{T}\mathcal{I}[\mu , R_{\theta }]$, where $R_{\theta }$ denotes rotation by angle θ (see Sect. 4). The resulting $\mathcal{T}\mathcal{I}$ curve is shown above. Local minima and maxima of the curve identify approximate symmetries and maximal asymmetries, respectively. For example, $R_{(34\pi /180)}$ is the maximal asymmetry associated with the local maximum at $\theta = 34\pi /180$ (blue diamond), while $R_{(62\pi /180)}$ is the approximate symmetry associated with the local minimum at $\theta = 62\pi /180$ (green triangle). The corresponding rotated images (bottom) demonstrate low overlap for the maximal asymmetry and high overlap for the approximate symmetry

*(Approximate Symmetries and Maximal Asymmetries)* Let $\{T_a\}_{a\in \mathcal {A}}$ be transformations as introduced in Definition 1. Suppose that $\mathcal {A}$ is a topological space. Then $T_s$ is an approximate symmetry (respectively, maximal asymmetry) if *s* is a local minimum (respectively, maximum) of $\mathcal{T}\mathcal{I}[\mu ,T_a]$ as a function of *a* (Fig. 1).

### Remark 6

For isometries $T_a: D \rightarrow D$, approximate symmetries are generalizations of exact symmetries because $\mathcal{T}\mathcal{I}[\mu ,T_a] \ge 0$ and $\mathcal{T}\mathcal{I}[\mu ,T_a]=0$ if and only if $\mu =T_a\mu$.

### Remark 7

When thinking of $\mathcal{T}\mathcal{I}[\mu ,T_a]$ as a function of *a*, we will refer to the graph $\{(a,\mathcal{T}\mathcal{I}[\mu ,T_a])\}\subset \mathcal {A} \times \mathbb {R}_{\ge 0}$ as the $\mathcal{T}\mathcal{I}$ *curve* (Fig. 1).

### Remark 8

While approximate symmetries may themselves be a discrete set, the $\mathcal{T}\mathcal{I}$ curve encodes more information. The full $\mathcal{T}\mathcal{I}$ curve, rather than the approximate symmetries, is the primary object of our analysis, and the fPCA of Sect. 5 operates on these curves as elements of a function space.

The center of an object μ is important for considering reflections and rotations; we would like to rotate about the center and reflect across an axis that passes through the center. Heuristically, the center of an object is the point in the domain of μ from which we can observe the most symmetry. We define this formally below.

### Definition 4

*(Symmetry Center)* Consider the continuous function $\mu : D\rightarrow \mathbb {R}_{>0}$. Suppose that $D\subset \mathbb {R}^n$ is compact and that $\mu >\delta$ for some δ>0. Let $\mathcal{T}\mathcal{I}$ be defined as in Definition 1. Furthermore, assume that $\mu _c$ is defined as

2 $$\begin{aligned} \mu _c(x) ={\left\{ \begin{array}{ll} \mu (x-c) & x\in D\cap (D+c) \\ \delta & x\in D \setminus (D+c) \,. \end{array}\right. } \end{aligned}$$

Now, define

$$\begin{aligned} M_c = \{a\in \mathcal {A}: a \text { is a local minimum of } \mathcal{T}\mathcal{I}[\mu _c,T_a]\}, \end{aligned}$$

and let

$$\begin{aligned} \overline{M}_c= M_c\setminus \{a\in \mathcal {A}: T_a =\text {id}\}. \end{aligned}$$

Then the symmetry center of μ is given by

3 $$\begin{aligned} \overline{x}:=\text {argmin}_{c\in D}\Bigg \{\inf _{a\in \overline{M}_c}\mathcal{T}\mathcal{I}[\mu _c,T_a]\Bigg \}. \end{aligned}$$

**Fig. 2:**
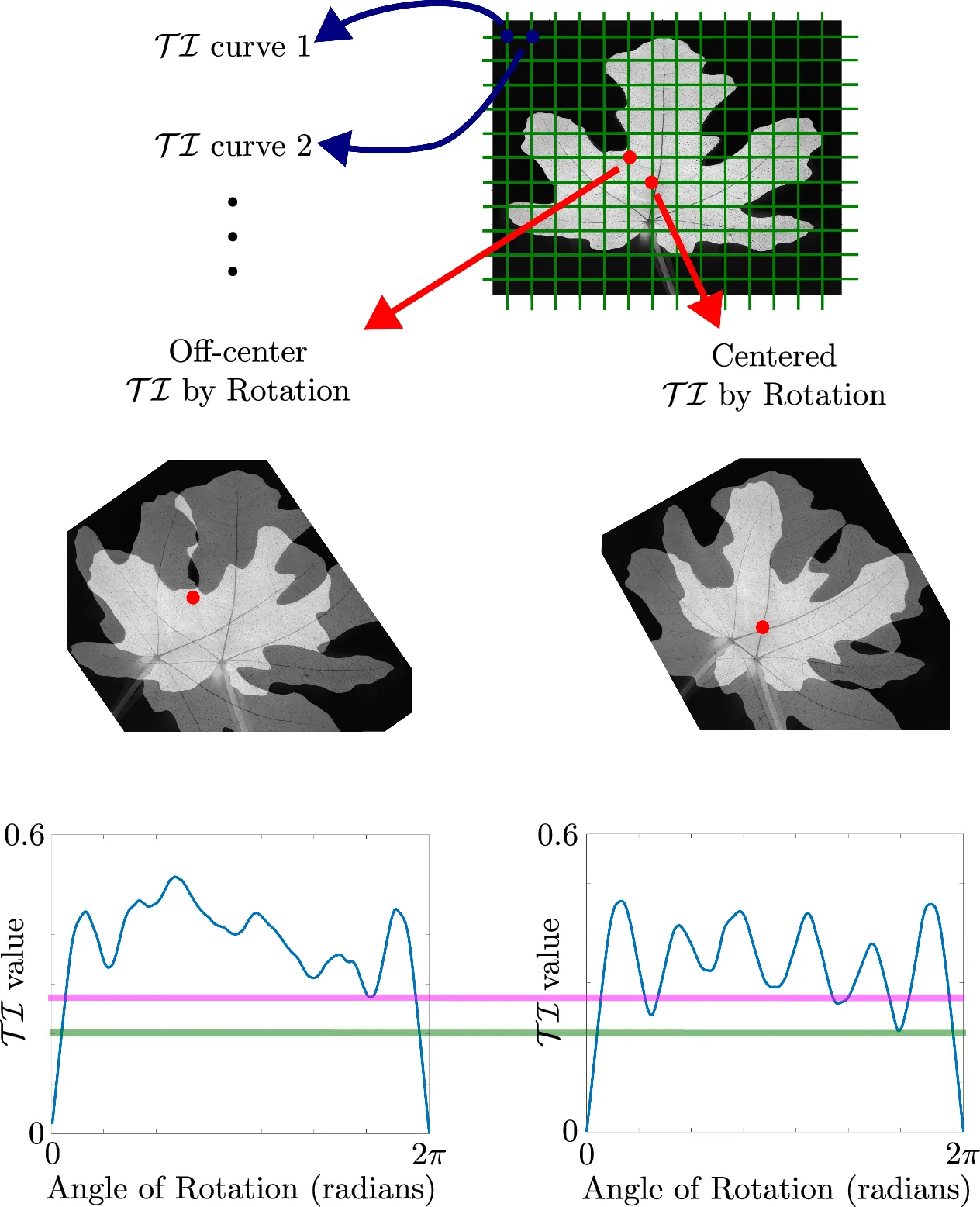
To locate the symmetry center, we sample a grid of candidate points in the domain (shown in green). For each grid point, we compute the corresponding $\mathcal{T}\mathcal{I}$ curve as if that point were the origin (see Sect. 4); two examples are shown below. We then record the smallest local minimum (excluding ones that correspond to the identity symmetry) for each potential symmetry center point. The point producing the smallest local minimum is then selected as the symmetry center of the image, shown by the red dot in the middle-right panel. In this case, the $\mathcal{T}\mathcal{I}$ curve in the bottom right plot attains the smallest local minimum (green). For comparison, the $\mathcal{T}\mathcal{I}$ curve obtained by rotating about the red point on the middle-left image has a higher minimum (pink)

In our computational pipeline, we use Definition 4 to identify the symmetry center of each image, finding the point from which we can observe the smallest $\mathcal{T}\mathcal{I}$ value (see Fig. 2). However, there are various ways to define the “most symmetry.” For example, one may consider the symmetry center to be the point with the largest number of approximate symmetries, with the lowest average $\mathcal{T}\mathcal{I}$, or with the smallest min to max ratio. In our simulations, we find that each of these notions finds approximately the same symmetry center of the object.

We note one limitation of Definition 4 in practice: because $\mathcal{T}\mathcal{I}$ is computed on $\widetilde{D} = D \cap T_a(D)$, candidate centers that produce little overlap between *D* and $T_a(D)$ under some transformation are evaluated on a smaller domain and can therefore attain spuriously small $\mathcal{T}\mathcal{I}$ values, biasing the argmin in (3) towards such candidates. This is the same concern discussed in Sect. A.1, where we provide an alternative definition of $\mathcal{T}\mathcal{I}$ that bypasses this issue but implies a slightly different notion of “symmetry." We show that the alternative definition of $\mathcal{T}\mathcal{I}$ improves center estimation in Sect. A.3.

Another natural alternative is the intensity-weighted mean or centroid of the pixel coordinates

4 $$\begin{aligned} \overline{x}:= \frac{\sum _{x\in D} x \mu (x)}{\sum _{x\in D} \mu (x)}, \end{aligned}$$

which is far cheaper to compute. In our dataset it lies close to the symmetry centers obtained from Definition 4, and may be a reasonable substitute when computational cost is a concern (see Sect. A.3).

We nonetheless retain Definition 4 for our applications. The centroid measures the distribution of intensity rather than symmetry, so agreement is a property of these images rather than a general guarantee, and computing $\mathcal{T}\mathcal{I}$ about the centroid yields strictly larger minima than computing it about the symmetry center. The centroid also yields a single point regardless of the symmetry transformation family. By contrast, the symmetry center locates the point from which the object exhibits the most symmetry and is adapted to the symmetry of interest.

### Remark 9

We assume that μ is continuous and *D* is compact to ensure that the argmin in (3) exists. In our application to image data, *D* represents a finite set of pixels, meaning that the argmin will exist despite μ being discontinuous.

### Remark 10

When searching for the symmetry center, we define $\overline{M}_c$ so that we only consider transformations $T_a$ that are not equal to the identity map, $\text {id}$. If instead we considered $M_c$ such that $T_a=\text {id}$ for some $a\in M_c$, then for every *c*, we have that

$$\begin{aligned} \min _{a\in M_c} \mathcal{T}\mathcal{I}[\mu _c,T_a]&=\mathcal{T}\mathcal{I}[\mu _c,\text {id}]=0. \end{aligned}$$

implying that every c∈D is a symmetry center, which is not useful for our analysis.

## Mathematical Properties of $\mathbf {\mathcal{T}\mathcal{I}}$

A central requirement for any symmetry measure is that it should capture intrinsic structure rather than arbitrary choices in how an object or image is presented. In our context, this means that $\mathcal{T}\mathcal{I}$ should be robust to affine transformations of the object of interest such as translation, rotation, reflection, and rescaling. This is especially important when analyzing large datasets, since inspecting each image individually is impractical. In this section, we show that two images representing the same object, but taken at different positions, orientations, or sizes produce $\mathcal{T}\mathcal{I}$ curves that only differ in predictable, well-characterized ways. For example, when $\{T_a\}$ denotes rotations by angle *a*, rotating an image does not change the $\mathcal{T}\mathcal{I}$ curve despite the change in pixel arrangement. This property allows $\mathcal{T}\mathcal{I}$ to reliably detect symmetries of images without requiring that they be perfectly aligned or scaled.

**Fig. 3:**
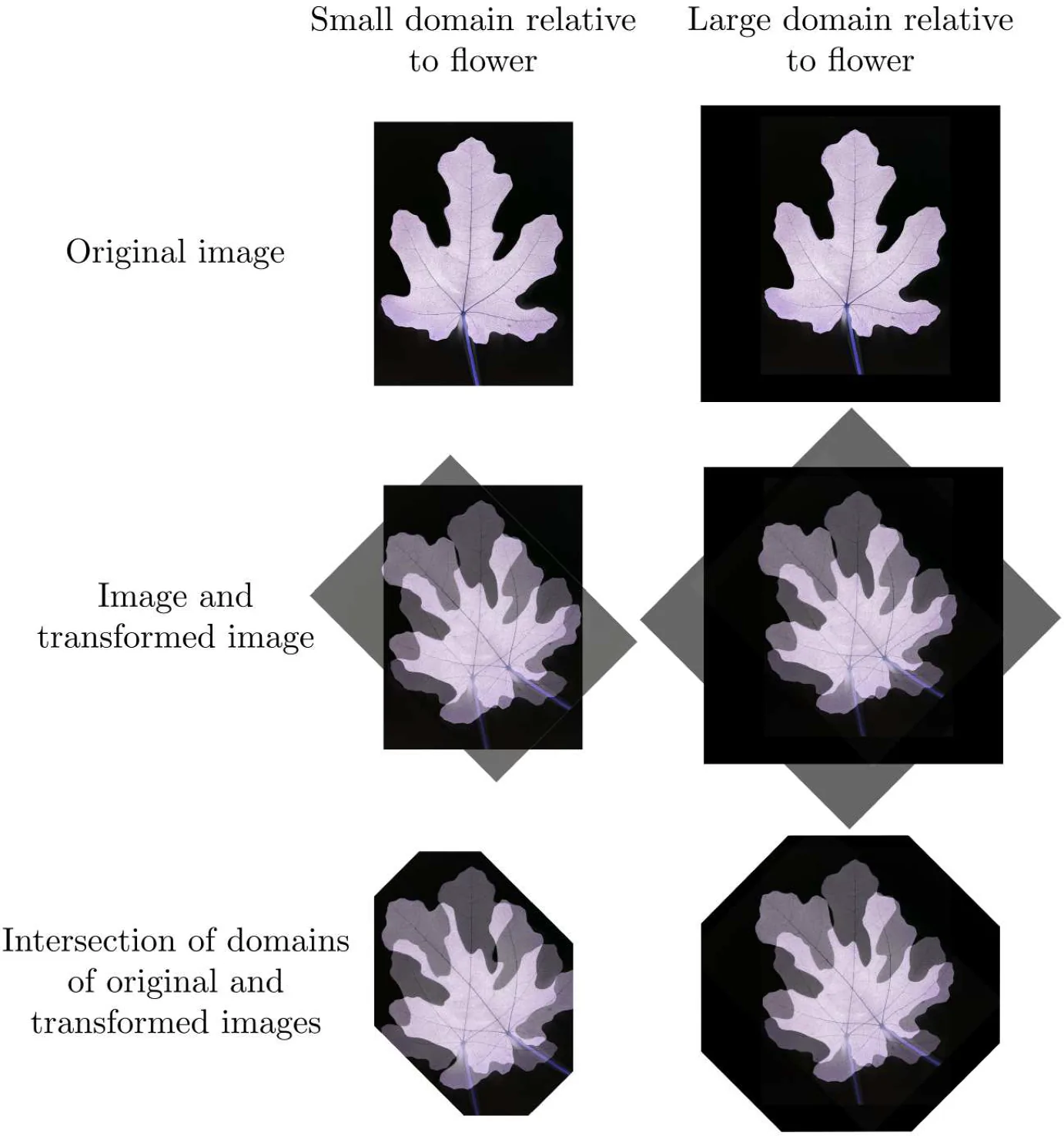
Schematic illustrating Proposition 1. Left: when the domain is small relative to the leaf, the set $\widetilde{D}$ (bottom left) cuts off parts of the leaf, unlike in $\mathbb {R}^2$. Right: extending the uniform background ensures $\widetilde{D}$ contains both the original and transformed leaf; since the overlapping background contributes 0 to $\mathcal{T}\mathcal{I}$, Proposition 1 shows that the $\mathcal{T}\mathcal{I}$ results match those of a function defined on all of $\mathbb {R}^2$

As before, to show that $\mathcal{T}\mathcal{I}$ is robust, we assume that our object of interest μ is a measurable, real-valued function on $\mathbb {R}^n$, bounded below by some positive constant δ. The results, however, hold for objects or images defined on $D\subsetneq \mathbb {R}^n$ if there is a sufficiently large uniform background, as proven below (Proposition 1, Fig. 3). Furthermore, although we prove all results for functions on $\mathbb {R}^n$, similar results hold for functions on $\mathbb {Z}^n$, such as image data (see Sect. 4.3).

In Proposition 1 below, *g*(*x*) defined on *F* represents the object of interest while the constant function *m* on $\mathbb {R}^n\setminus F$ represents the uniform background. If a domain *D* satisfies the specified properties, then $\mathcal{T}\mathcal{I}$ is the same whether considering μ on the entire space or restricted to *D*.

### Proposition 1

(Large background domain makes $\mathcal{T}\mathcal{I}$ robust) Let $\mu : \mathbb {R}^n\rightarrow \mathbb {R}$ be defined as follows:

$$\begin{aligned} \mu (x) = {\left\{ \begin{array}{ll} g(x) & x\in F \\ m & x\in \mathbb {R}^n \setminus F \end{array}\right. } \end{aligned}$$

for some $F\subset \mathbb {R}^n$ and some constant m>0. Let $\{T_a: \mathbb {R}^n \rightarrow \mathbb {R}^n\}_{a\in \mathcal {A}}$ arbitrary family of invertible transformations. Furthermore, let $D\subset \mathbb {R}^n$ be such that F⊂D and $T_a^{\pm }(F)\subset D$ for all $a\in \mathcal {A}$. Then

$$\begin{aligned} \mathcal{T}\mathcal{I}[\mu , T_a] = \mathcal{T}\mathcal{I}\left[ \mu \big |_D,T_a\right] \ \text {for all} \ a\in \mathcal {A}. \end{aligned}$$

### Proof

Let $\widetilde{D} = D \cap T_a(D)$. Since F⊂D and $T_a^{\pm }(F)\subset D$ for all a, $\mu \big |_{\mathbb {R}^n\setminus \widetilde{D}} \equiv m$ and $(T_a\mu )\big |_{\mathbb {R}^n\setminus \widetilde{D}} \equiv m$. Therefore, we have that

$$\begin{aligned} \mathcal{T}\mathcal{I}[\mu ,T_a]&= \int _{\mathbb {R}^n} \mu \log \left( \frac{\mu }{T_a\mu }\right) \\&= \int _{\widetilde{D}} \mu \log \left( \frac{\mu }{T_a\mu }\right) + \int _{\mathbb {R}^n\setminus \widetilde{D}} \mu \log \left( \frac{\mu }{T_a\mu }\right) \\&=\int _{\widetilde{D}} \mu \log \left( \frac{\mu }{T_a\mu }\right) + \int _{\mathbb {R}^n\setminus \widetilde{D}} m \log \left( \frac{m}{m}\right) \\&= \int _{\widetilde{D}} \mu \log \left( \frac{\mu }{T_a\mu }\right) \\&= \mathcal{T}\mathcal{I}\left[ \mu \big |_D,T_a\right] . \ \end{aligned}$$

□

Since an object with a sufficiently large uniform background has the same $\mathcal{T}\mathcal{I}$ as an object defined on $\mathbb {R}^n$, we assume the object is defined on $\mathbb {R}^n$ for the rest of this section.

### Properties for General Transformations

To formally establish the robustness of $\mathcal{T}\mathcal{I}$, we analyze its behavior when a transformation *A* is applied to the object μ. We consider the general case where *A* is an affine transformation and may represent a combination of translation, rotation, and scaling. We show that the $\mathcal{T}\mathcal{I}$ curve of the transformed data Aμ is not an arbitrary new function, but a predictable mapping of the original. This allows us to prove that the core information about the symmetry is preserved despite changes in the object’s orientation or position.

The following identity characterizes how $\mathcal{T}\mathcal{I}$ changes when an affine transformation acts on μ. Transforming the object of interest μ transforms the $\mathcal{T}\mathcal{I}$ curve in a similar way. Loosely speaking, the “shape” of the $\mathcal{T}\mathcal{I}$ curve will stay the same when an affine transformation is applied to μ.

#### Remark 11

For an affine transformation $A\in \text {Aff}(n)$, with determinant as defined in Remark 5, the quantity |det(A)| is the factor by which *A* scales volumes; for any measurable set $E\subset \mathbb {R}^n$ with image $AE:=\{Ax: x\in E\}$, we have $\text {vol}(AE) = |\det (A)|\ \text {vol}(E)$. Writing $\chi _E$ for the indicator function of *E* and observing that $\chi _E(A^{-1}x)=\chi _{AE}(x)$, it follows that

$$\begin{aligned} \int _{\mathbb {R}^n} \chi _E(A^{-1}x)\ dx = |\det (A)| \int _{\mathbb {R}^n} \chi _E(x)\ dx. \end{aligned}$$

Approximating by simple functions extends the identity to any integrable $f:\mathbb {R}^n\rightarrow \mathbb {R}$, so that

5 $$\begin{aligned} \int _{\mathbb {R}^n} f(A^{-1}x)\ dx = |\det (A)| \int _{\mathbb {R}^n} f(x)\ dx. \end{aligned}$$

This is the change of variables x↦Ax applied in the proofs that follow, and it is the source of the factor |det(A)| in (6).

#### Proposition 2

($\mathcal{T}\mathcal{I}$ curve of transformed data is conjugate) Let $\mu : \mathbb {R}^n \rightarrow \mathbb {R}_{>0}$ be a function bounded below by some positive constant δ. Furthermore, let $A,B\in \text {Aff}(n)$. Then

6 $$\begin{aligned} \mathcal{T}\mathcal{I}[\mu ,B] = \frac{1}{|\det {A}|}\mathcal{T}\mathcal{I}[A\mu , ABA^{-1}]. \end{aligned}$$

#### Proof

$$\begin{aligned} \mathcal{T}\mathcal{I}[A\mu , ABA^{-1}]&= \int A\mu \log \left( \frac{A\mu }{ABA^{-1}A\mu }\right) \\&= \int \mu \circ A^{-1} \log \left( \frac{\mu \circ A^{-1}}{\mu \circ A^{-1}\circ A\circ B^{-1}\circ A^{-1}}\right) \\&= \int \mu \circ A^{-1} \log \left( \frac{\mu \circ A^{-1}}{\mu \circ B^{-1}\circ A^{-1}}\right) \\&= |\det (A)|\int \mu \log \left( \frac{\mu }{\mu \circ B^{-1}}\right) &  (x\mapsto Ax) \\&=|\det (A)| \mathcal{T}\mathcal{I}[\mu ,B]. \end{aligned}$$

□

Since the “shape" of the $\mathcal{T}\mathcal{I}$ curve is the same when an affine transformation acts on μ, some structure in the set of approximate symmetries of μ should also be preserved. We show below that transforming the data does not change the number of approximate symmetries. In fact, the set of approximate symmetries is conjugate.

#### Proposition 3

(Symmetries of transformed data are conjugate) Let $\mu :\mathbb {R}^n\rightarrow \mathbb {R}_{>0}$ be a function bounded below by some positive constant δ. Suppose $\{T_a\} = \text {Aff}(n)$ with the standard topology. If $\mathcal {S}\subset \text {Aff}(n)$ is the set of approximate symmetries of μ, then for any $A\in \text {Aff}(n)$, $A\mathcal {S}A^{-1}$ is the set of approximate symmetries of Aμ.

#### Proof

Let $M \in \text {Aff}(n)$ be an approximate symmetry of μ (Definition  3). Then by definition, there exists some neighborhood of *M*, call it $\mathcal {N}_M$, such that for all $B\in \mathcal {N}_M$, $\mathcal{T}\mathcal{I}[\mu ,M] \le \mathcal{T}\mathcal{I}[\mu ,B]$. Using (6), we can immediately see that for any $A\in \text {Aff}(n)$ and any $B\in \mathcal {N}_M$, we also have that

7 $$\begin{aligned} \mathcal{T}\mathcal{I}[A\mu ,AMA^{-1}] \le \mathcal{T}\mathcal{I}[A\mu ,ABA^{-1}]. \end{aligned}$$

Consider the neighborhood of $AMA^{-1}$ given by $\overline{\mathcal {N}}=A\mathcal {N}_MA^{-1}$. By (7), for every $C \in \overline{\mathcal {N}}$ we have that

8 $$\begin{aligned} \mathcal{T}\mathcal{I}[A\mu ,AMA^{-1}] \le \mathcal{T}\mathcal{I}[A\mu ,C]. \end{aligned}$$

Since there exists a neighborhood of $AMA^{-1}$ given by $\overline{\mathcal {N}}$ such that for every element $C \in \overline{\mathcal {N}}$, (8) holds, $AMA^{-1}$ is an approximate symmetry of Aμ by definition. Since this holds $\forall M \in \text {Aff}(n)$, we have shown that $A\mathcal {S}A^{-1}$ is a subset of the approximate symmetries of Aμ. We can prove the reverse inclusion in the same manner. □

We end this section by showing that scaling μ results in scaling the $\mathcal{T}\mathcal{I}$ curve and thus does not change the set of approximate symmetries.

#### Proposition 4

(Scaling an object scales $\mathcal{T}\mathcal{I}$) Let $\mu :\mathbb {R}^n\rightarrow \mathbb {R}_{>0}$ be a function bounded below by some positive constant δ, and consider the scaling matrix S=sI for s≠0, where *I* is the n×n identity matrix. Then for any $A\in \text {Aff}(n)$,

$$\begin{aligned} \mathcal{T}\mathcal{I}[S\mu ,A] = s^n\mathcal{T}\mathcal{I}[\mu ,A] \end{aligned}$$

#### Proof

$$\begin{aligned} \mathcal{T}\mathcal{I}[S\mu ,A]&= \int S\mu \log \left( \frac{S\mu }{AS\mu }\right) \\&= \int \mu \circ S^{-1} \log \left( \frac{\mu \circ S^{-1}}{\mu \circ S^{-1}\circ A^{-1}}\right) \\&= |\det (S)| \int \mu \log \left( \frac{\mu }{\mu \circ S^{-1} \circ A^{-1} \circ S}\right) &  (x \mapsto Sx) \\&=s^n \int \mu \log \left( \frac{\mu }{\mu \circ A^{-1}}\right) &  \text {(}S\text { commutes)} \\&= s^n \mathcal{T}\mathcal{I}[\mu ,A]. \end{aligned}$$

□

Collectively, these results demonstrate that while affine transformations may shift or scale the data, the resulting $\mathcal{T}\mathcal{I}$ curves and their approximate symmetries remain conjugate to the original. This mathematical consistency ensures that the intrinsic symmetry of an object, as measured by $\mathcal{T}\mathcal{I}$, is preserved under deformation, providing a reliable foundation for empirical analysis. Building on this general framework, we now focus on rotational and reflectional symmetries, which are particularly important in biological systems.

### Properties for Rotations and Reflections

In most biological applications, we are interested in rotational and reflectional symmetries given by the transformations $\{R_{\theta }\}$ for $\theta \in [0,2\pi )$ and $\{\kappa _{\theta }\}$ for $\theta \in [0,\pi )$ (with the subspace topology inherited from $\mathbb {R}$). In homogeneous coordinates (Remark 5), reflections and rotations in $\mathbb {R}^2$ take the form

9 $$\begin{aligned} R_{\theta }= \begin{pmatrix} \cos (\theta ) & -\sin (\theta ) & 0\\ \sin (\theta ) & \cos (\theta ) & 0 \\ 0 & 0 & 1 \end{pmatrix} \,, \end{aligned}$$

10 $$\begin{aligned} \kappa _{\theta } = \begin{pmatrix} \cos (2\theta ) & \sin (2\theta ) & 0 \\ \sin (2\theta ) & -\cos (2\theta ) & 0 \\ 0 & 0 & 1 \end{pmatrix}. \end{aligned}$$

Note that reflections only need to be defined for $\theta \in [0,\pi )$ because $\kappa _{\theta }=\kappa _{\theta +\pi }$.

Considering these transformations leads to special invariance and equivariance properties of the $\mathcal{T}\mathcal{I}$ curve when we reorient the object μ by taking $R_{\varphi }\mu$ or $\kappa _{\varphi }\mu$. To describe these properties, we fix a function μ and refer to the $\mathcal{T}\mathcal{I}$ curves of $\mathcal{T}\mathcal{I}[\mu ,R_{\theta }]$ and $\mathcal{T}\mathcal{I}[\mu ,\kappa _{\theta }]$ by $\mathcal{T}\mathcal{I}_{\text {rot}}(\theta )$ and $\mathcal{T}\mathcal{I}_{\text {ref}}(\theta )$, respectively. We also periodically extend $\mathcal{T}\mathcal{I}_{\text {ref}}(\theta )$ so that both $\mathcal{T}\mathcal{I}$ curves are defined on [0,2π).

Next, we define the operators

11 $$\begin{aligned}&F_{\text {rot}}: \mu \mapsto \mathcal{T}\mathcal{I}_{\text {rot}}(\theta ) \,, \end{aligned}$$

12 $$\begin{aligned}&F_{\text {ref}}: \mu \mapsto \mathcal{T}\mathcal{I}_{\text {ref}}(\theta ). \end{aligned}$$

Lastly, define the following transformations on [0,2π):

13 $$\begin{aligned} \overline{R}_{\varphi }(\theta )&=\theta + \varphi \mod 2\pi \,, \end{aligned}$$

14 $$\begin{aligned} \overline{\kappa }_{\varphi }(\theta )&=2\varphi -\theta \mod 2\pi , \end{aligned}$$

and as before denote the transformed versions of the $\mathcal{T}\mathcal{I}$ curves as

$$\begin{aligned} \overline{R}_{\varphi }\mathcal{T}\mathcal{I}(\theta )&:= \mathcal{T}\mathcal{I}\left( \overline{R}_{\varphi }^{-1}(\theta )\right) \,, \\ \overline{\kappa }_{\varphi }\mathcal{T}\mathcal{I}(\theta )&:= \mathcal{T}\mathcal{I}\left( \overline{\kappa }_{\varphi }^{-1}(\theta )\right) . \end{aligned}$$

See Fig. S1 for examples of these transformations. We then have the following result on how $\mathcal{T}\mathcal{I}$ commutes with rotation and reflection transformations.

#### Proposition 5

(Equivariance of $F_{\text {rot}}$ and $F_{\text {ref}}$ under rotation and reflection of μ) Let $\mu :\mathbb {R}^2 \rightarrow \mathbb {R}_{>0}$ be some function bounded below by a positive constant δ. Furthermore, let $F_{\text {rot}}$, $F_{\text {ref}}$, $\overline{R}_{\varphi }$, and $\overline{\kappa }_{\varphi }$ be defined as in (11)-(14). Then the following equations hold for every $\varphi \in [0,2\pi )$.

15 $$\begin{aligned} F_{\text {rot}}(R_{\varphi } \mu )&= F_{\text {rot}}(\mu ) \end{aligned}$$

16 $$\begin{aligned} F_{\text {rot}}(\kappa _{\varphi }\mu )&= \overline{\kappa }_0F_{\text {rot}}(\mu ) \end{aligned}$$

17 $$\begin{aligned} F_{\text {ref}}(R_{\varphi }\mu )&= \overline{R}_{\varphi }F_{\text {ref}}(\mu ) \end{aligned}$$

18 $$\begin{aligned} F_{\text {ref}}(\kappa _{\varphi }\mu )&=\overline{\kappa }_{\varphi }F_{\text {ref}}(\mu ) . \end{aligned}$$

It follows that:

1. If $\mathcal {S}$ is the set of rotational approximate symmetries of μ, then $\mathcal {S}$ is the set of rotational approximate symmetries for $R_{\varphi }\mu$.
2. If $\mathcal {S}$ is the set of rotational approximate symmetries of $\kappa _{\varphi }\mu$, then $\overline{\kappa }_0\mathcal {S}$ is the set of rotational approximate symmetries for $\kappa _{\varphi }\mu$.
3. If $\mathcal {S}$ is the set of reflectional approximate symmetries of μ, then $\overline{R}_{\varphi }\mathcal {S}$ is the set of reflectional approximate symmetries of $R_{\varphi } \mu$
4. If $\mathcal {S}$ is the set of reflectional approximate symmetries of μ, then $\overline{\kappa }_{\varphi }\mathcal {S}$ is the set of reflectional approximate symmetries of $\kappa _{\varphi }\mu$.

**Fig. 4:**
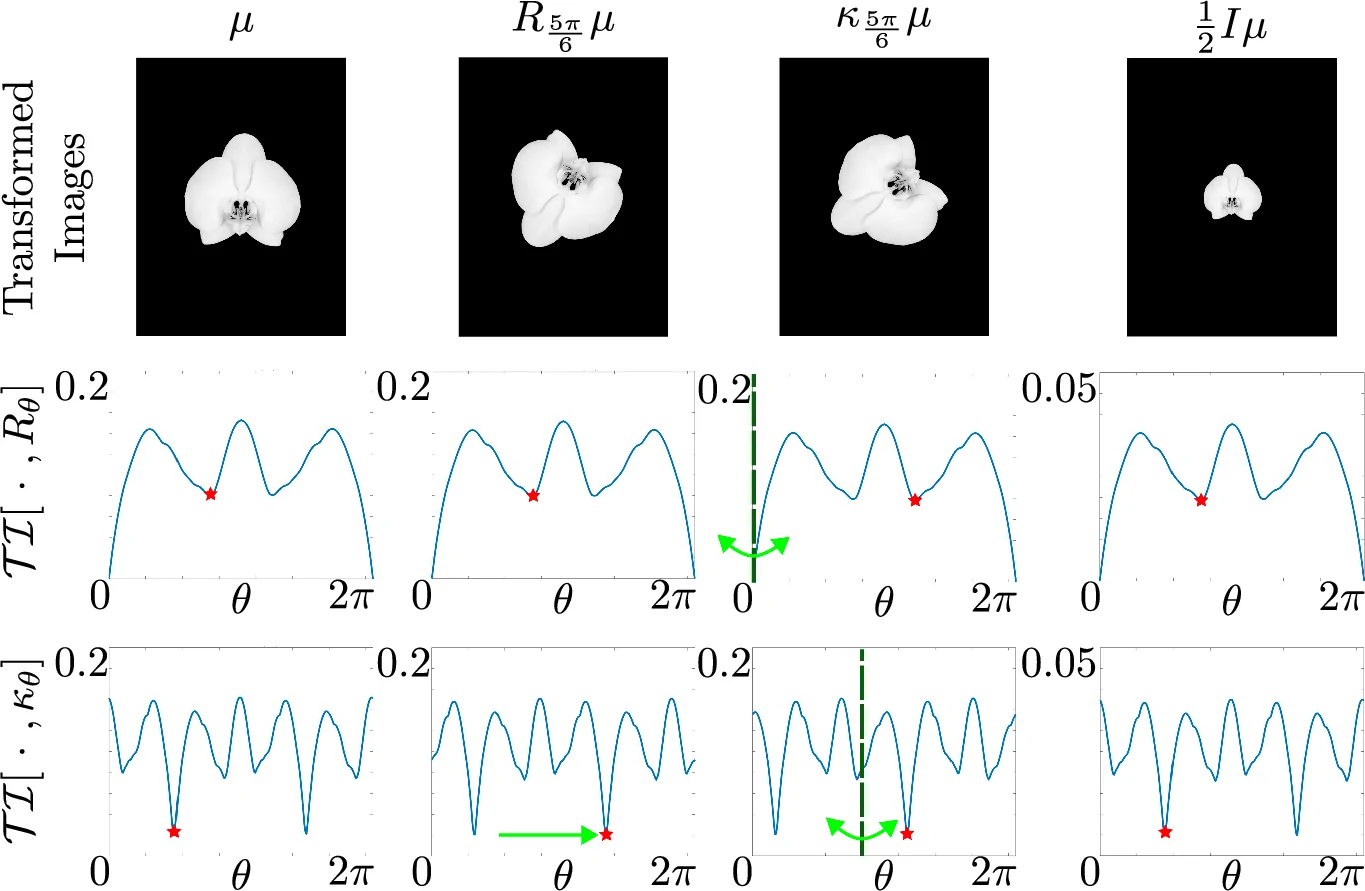
Effect of image transformations on $\mathcal{T}\mathcal{I}$. In row 2 (rotational symmetries), the $\mathcal{T}\mathcal{I}$ curve is invariant under rotation (col. 2), reflected across θ=0 modulo 2π (green line) under image reflection (col. 3), and scaled by $(1/2)^2$ under image scaling (col. 4). In row 3 (reflection symmetries), rotation by 5π/6 translates the $\mathcal{T}\mathcal{I}$ curve by the same amount (col. 2), reflection across $\theta = 5\pi /6$ the curve across that axis modulo 2π (green line) (col. 3), and scaling again multiplies the $\mathcal{T}\mathcal{I}$ curve by $(1/2)^2$ (col. 4). Red stars highlight points that help visualize the transformations. Small variations in $\mathcal{T}\mathcal{I}$ curves arise from numerical error and boundary effects

Figure 4 shows an illustration of Proposition 5 for a flower image. In Sect. A.2, we provide the proofs for these statements.

## Computational Pipeline

We now describe our MATLAB computational pipeline for calculating the transformation information of image data. The pipeline consists of the following steps, which we explain in further detail in this section:

1. Preprocessing images.
2. Representing an image with a real-valued function $\overline{\mu }$ on a finite domain.
3. Computing $\mathcal{T}\mathcal{I}$ of image data.
4. Centering $\overline{\mu }$ to obtain a new function μ.

### Preprocessing Images

$\mathcal{T}\mathcal{I}$ is designed to reliably extract symmetry information with minimal user input. Common imaging techniques, such as fluorescence microscopy or slide imaging, provide the uniform backgrounds necessary for high-fidelity analyses, meaning $\mathcal{T}\mathcal{I}$ can often be applied directly to raw data.

In our applications (Sect. 5), we ensure that the images have uniform black backgrounds through either controlled imaging conditions or straightforward digital background removal. In cases where images have light backgrounds or backgrounds with significant variations in pixel intensity, simple preprocessing steps such as taking the negative, smoothing, or thresholding can be employed to isolate the object of interest. These minor adjustments ensure that the resulting $\mathcal{T}\mathcal{I}$ curves faithfully reflect the symmetry of the data. For further details on these implementations, see Sect. A.4 and Fig. S2.

### Representing Each Image with A Real-Valued Function $\overline{\mu }$ on a Finite Domain

An image can be represented as a an array of three $\ell \times w$ matrices *R*, *G*, and *B* that represent the intensities of red, green, and blue light at each pixel on a scale from 0 to 255. We convert an image to grayscale by taking a weighted sum of *R*, *G*, and *B*. The result is a single grayscale matrix $M=(m_{ij})$ with $0\le m_{ij}\le 255$ for any *i*, *j*. We define the representative function for the image as

19 $$\begin{aligned} \overline{\mu }(i,j)=m_{ij}+\delta \end{aligned}$$

for some δ>0 (Fig. S2). We add a small δ value to each image to ensure that the function is positive on its domain, as is required to compute $\mathcal{T}\mathcal{I}$. By Proposition 6 (Lipshitz continuity of $\mu \mapsto \mathcal{T}\mathcal{I}[\mu ,T_a]$), adding a small δ will only change the $\mathcal{T}\mathcal{I}$ curve by a small amount. In our applications to flowers and ferns, we take δ=1. Note that the domain *D* of $\overline{\mu }$ is the finite set

20 $$\begin{aligned} D = \{i: 1\le i \le \ell \} \times \{j: 1\le j\le w\}. \end{aligned}$$

#### Remark 12

The weighted sum of *R*,*G*, and *B* matrices can be adjusted to emphasize certain colors. For example, Gandhi et al. (2021) emphasize green by taking the brightness of green minus the average pixel brightness (M=(2G-R-B)/3) when analyzing approximate symmetries of the unicellular freshwater desmid *Micrasteria*, which is a type of green algae. In Fig. S3, we demonstrate this method for an image of a leaf on dirt.

### Computing $\mathcal{T}\mathcal{I}$ of Image Data

**Fig. 5:**
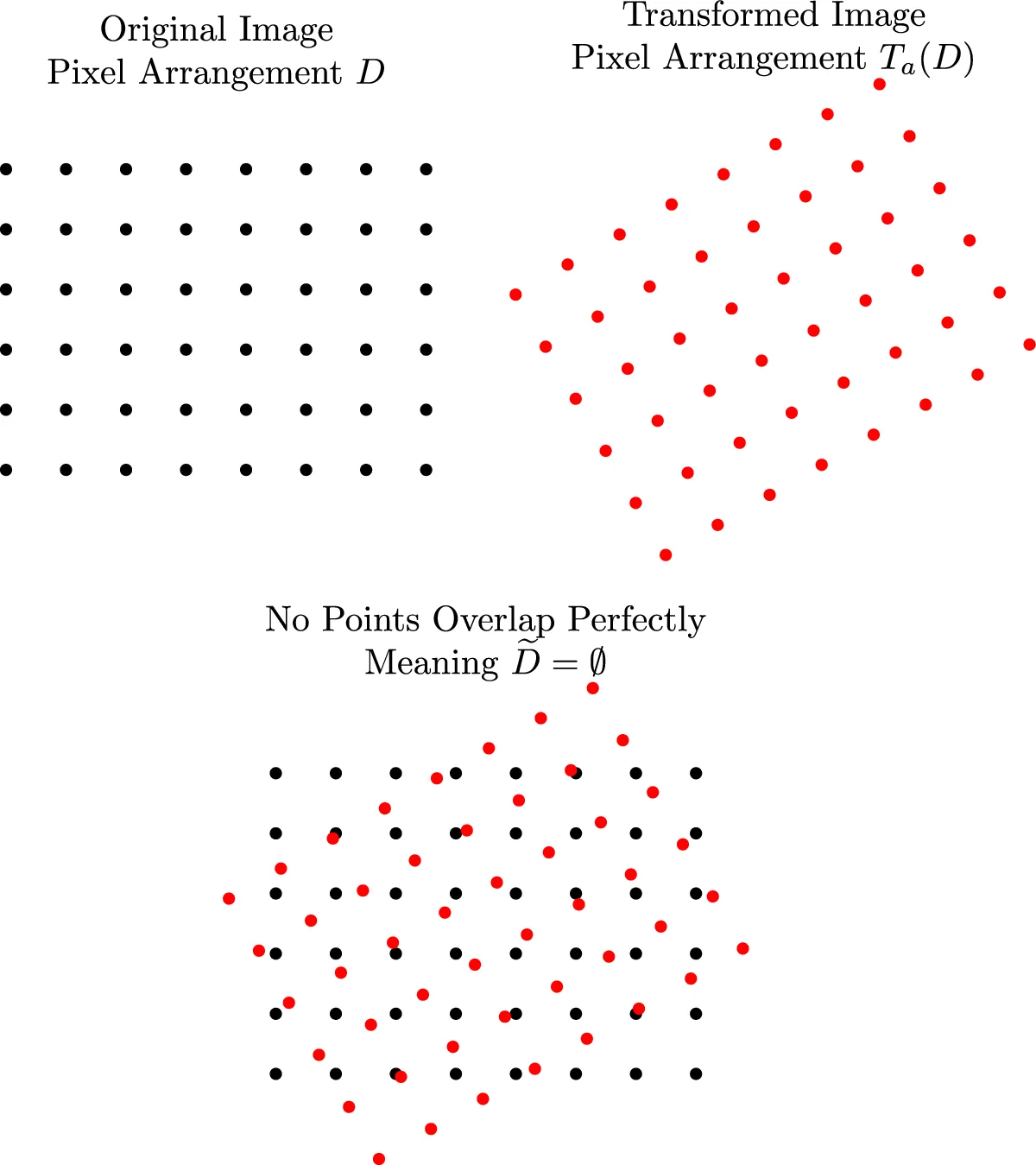
An image $\overline{\mu }$ as in Sect. 4.2 is supported on a lattice of pixels *D* (top left). Thus, the transformed image $T_a\overline{\mu }$ is supported on a transformed domain $T_a(D)$ (top right). Taking the intersection of these domains (bottom), no pixels align perfectly, so $\widetilde{D} = D \cap T_a(D) = \emptyset$

In computing the $\mathcal{T}\mathcal{I}$ of image data, we note that a grayscale image $\overline{\mu }$ (19) is defined on a finite domain *D*; therefore, the set $\widetilde{D}=D\cap T_a(D)$ will almost always be empty (see Fig. 5), and $\mathcal{T}\mathcal{I}$ will thus be zero. To avoid this issue, we redefine $T_a \overline{\mu }$ on a subset of the lattice of integers by using linear interpolation of $\overline{\mu }$ between the points in $T_a(D)$. For the remainder of this manuscript, we will abuse notation and assume that we have already performed this process when considering the transformed image $T_a\overline{\mu }$, so that it is defined on a set that we denote as $\widetilde{T}_a(D)\subset \mathbb {Z}^2$. Then, for the application to images, we can rewrite $\mathcal{T}\mathcal{I}$ as

$$\begin{aligned} \mathcal{T}\mathcal{I}[\overline{\mu },T_a] =\sum _{(i,j)\in \widetilde{D}} \overline{\mu }(i,j) \log \left( \frac{\overline{\mu }(i,j)}{T_a\overline{\mu }(i,j)}\right) \end{aligned}$$

where $$\widetilde{D}=D\cap \widetilde{T}_a(D)$$.

### Centering $\overline{\mu }$ to Obtain a New Function μ

To analyze an image’s invariance under rotations and reflections, we must first establish its symmetry center (Definition 4). Given a grayscale image represented as a function $\overline{\mu }: D \rightarrow \mathbb {R}$ (Sect. 4.2) and a set of transformations $\{T_a\}$, the symmetry center can be found using Definition 4.

Numerically, we begin by selecting a grid of candidate symmetry centers c∈D. Candidate centers are restricted to the central 25% of the image since candidates near the boundary produce trivial symmetries (Sect. 2, Fig. 11). For each candidate, we translate the image so that *c* is mapped to the origin, obtaining the centered image $\overline{\mu }_c$. Fixing the center, we compute transformation information $\mathcal{T}\mathcal{I}[\overline{\mu }_c,T_a]$ and find the approximate symmetries, excluding the identity symmetry. The strongest approximate symmetry, given by the smallest local minimum of the $\mathcal{T}\mathcal{I}$ curve, is stored (Fig. 2). Repeating this procedure over all candidate symmetry centers *c* produces a scalar value for each candidate. The true symmetry center is then defined as the point *c* at which this value achieves its minimum. We then set $\mu :=\overline{\mu }_c$ to be the final function for our analysis. Details of the implementation, including discretization choices, and peak-detection procedures are provided in the accompanying code repository (GitHub 2025).

In our applications, we compute the symmetry center for two distinct transformation groups: reflections $\{\kappa _{\theta }\}$ across an axis at angle θ, and rotations $\{R_{\theta }\}$ by angle θ (9),(10). We denote the resulting reflectional symmetry center as $\overline{x}^{\kappa }$ and the rotational symmetry center as $\overline{x}^R$. Our pipeline produces four specific $\mathcal{T}\mathcal{I}$ curves:

1. Rotation about $$\overline{x}^R$$: $$\mathcal{T}\mathcal{I}[\overline{\mu }_{\overline{x}^R}, R_{\theta }]$$.
2. Reflection about $$\overline{x}^R$$: $$\mathcal{T}\mathcal{I}[\overline{\mu }_{\overline{x}^R}, \kappa _{\theta }]$$.
3. Rotation about $$\overline{x}^{\kappa }$$: $$\mathcal{T}\mathcal{I}[\overline{\mu }_{\overline{x}^{\kappa }}, R_{\theta }]$$.
4. Reflection about $$\overline{x}^{\kappa }$$: $$\mathcal{T}\mathcal{I}[\overline{\mu }_{\overline{x}^{\kappa }}, \kappa _{\theta }]$$.

#### Remark 13

As a consequence of Proposition 7, if an object has perfect reflection symmetry, then the $\mathcal{T}\mathcal{I}$ curves for rotations and reflections contain identical information.

## Applications

We apply the computational framework outlined in Sect. 4 to two datasets of flower and respectively fern images. The resulting collection of $\mathcal{T}\mathcal{I}$ curves characterizes the symmetries present in each image, enabling the use of functional data analysis to explore correlations between symmetry and specific functional properties of the specimens. As an initial step, we project each image’s $\mathcal{T}\mathcal{I}$ curve onto the first two functional principal components (i.e. *functional principal component analysis* (fPCA)) using the PACE toolbox (Yao et al. 2003) for the flower $\mathcal{T}\mathcal{I}$ curves and Shi et al. (2022)’s code for the fern $\mathcal{T}\mathcal{I}$ curves. We use two methods as each is optimized for functions on $\mathbb {R}$ and $\mathbb {R}^2$, respectively.

**Fig. 6:**
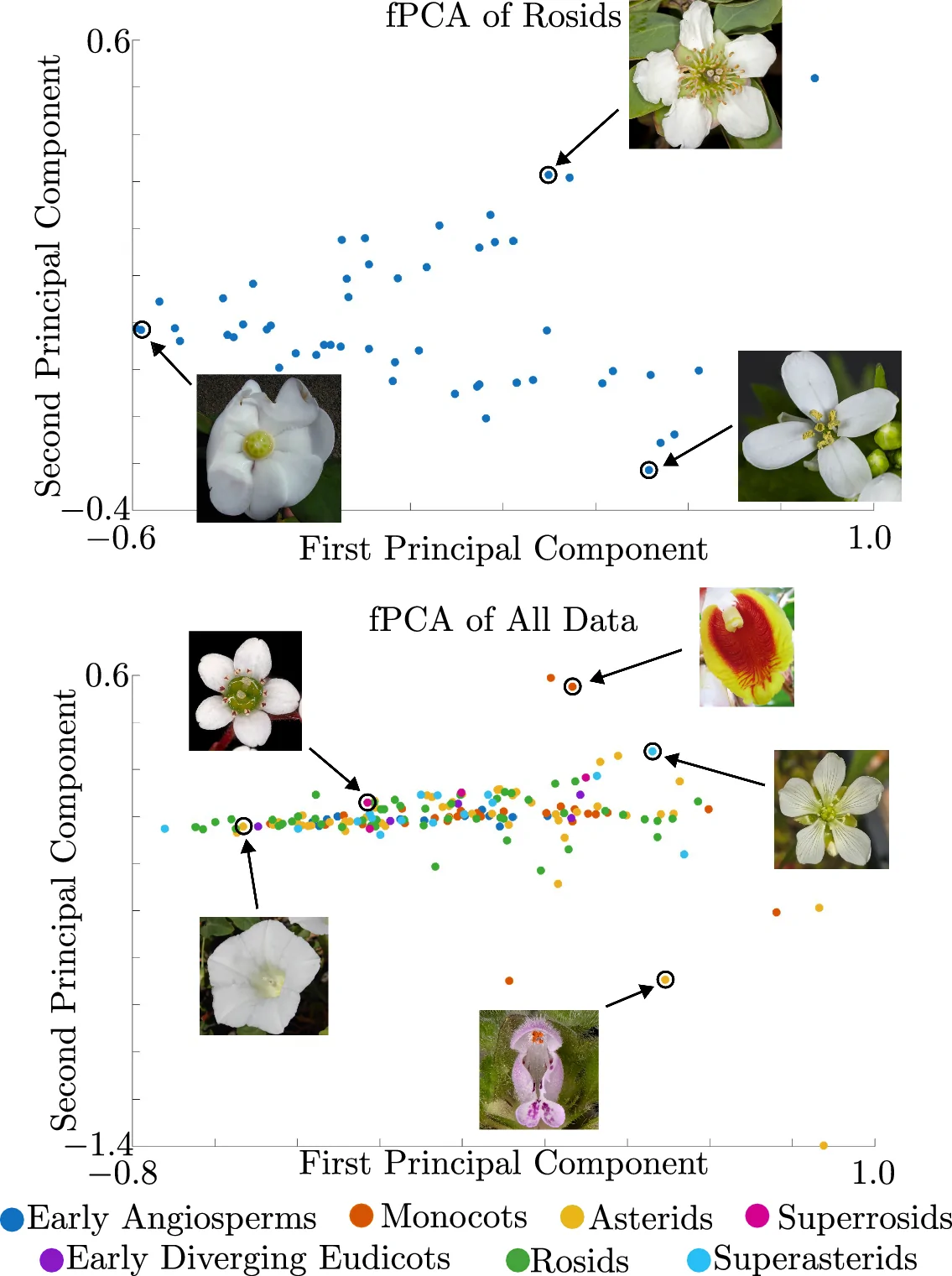
fPCA of $\mathcal{T}\mathcal{I}$ curves by rotation about the rotational symmetry center ($\mathcal{T}\mathcal{I}[\mu _{\overline{x}^R},R_{\theta }]$) reveals a continuous landscape of symmetries. Top: rosid curves projected onto the first two principal components, capturing 85.9% of the variance. Two branches emerge corresponding to 5-fold and 4-fold symmetries. Where the branches converge, flowers have indistinct petals and small variations in their $\mathcal{T}\mathcal{I}$ curves. Bottom: all projected $\mathcal{T}\mathcal{I}$ curves, color-coded by clade, capturing 71.9% of the variance. Flowers with radial symmetry cluster along a central axis, transitioning from indistinct to well-defined petals from left to right, while flowers off the axis exhibit bilateral symmetry. The flower images presented on the graphs are stock images that closely match the image data used for our analysis (Descouens 2022; Game 2017; Starr and Starr 2026; North Queensland Plants 2026; Reese 2024; falcon_311 2025; Carr 2005; Edison and Ford Winter Estates 2010; Smith 2024)

In the floral dataset, the fPCA plots (Fig. 6) reveal that $\mathcal{T}\mathcal{I}$ curves overlap across evolutionarily distinct clades, suggesting that symmetry types evolved independently and are therefore functionally significant. In the fern dataset, we investigate fractal symmetry, demonstrating that $\mathcal{T}\mathcal{I}$ can represent arbitrary symmetry transformations. In both applications, the visualizations demonstrate that the symmetries are distributed continuously in the function space of $\mathcal{T}\mathcal{I}$ curves, rather than discrete clusters based on idealized symmetry categories.

### Floral Symmetries

We begin by investigating the symmetries of flowers from the *Plant Gateway* poster (Byng et al. 2018). Using the computational pipeline described in Sect. 4, we generate $\mathcal{T}\mathcal{I}$ curves by rotation and reflection for each image. We then perform fPCA and present the results for rotational $\mathcal{T}\mathcal{I}$ in Fig. 6. The analogous results for reflectional $\mathcal{T}\mathcal{I}$ are included in Sect. A.5. For our dataset, the remaining two $\mathcal{T}\mathcal{I}$ curves ($\mathcal{T}\mathcal{I}[\overline{\mu }_{\overline{x}^R},\kappa _{\theta }]$ and $\mathcal{T}\mathcal{I}[\overline{\mu }_{\overline{x}^{\kappa }},R_{\theta }]$, Sect. 4.4) are redundant since the rotational center $\overline{x}^R$ and reflectional center $\overline{x}^{\kappa }$ agree closely (Sect. A.3). The rotational fPCA results show that floral symmetry exists along a continuum rather than within the traditional discrete categories of bilateral (zygomorphic) and radial (actinomorphic) symmetries. Notably, we see in the bottom panel of Fig. 6 that points with different colors, representing flowers from different clades, are intermixed. This shows that the range of exhibited symmetries are represented within each clade, providing evidence that the symmetries evolved independently and are not merely incidental but adaptive. We come to the same conclusion by analyzing reflectional symmetry (Sect. A.5).

The fPCA also effectively captures intuitive morphological differences; for example, the fPCA of rosids presents two diverging branches. Flowers towards the tip of the upper branch have 5-fold rotational symmetry, while those at the tip of the lower branch have 4-fold rotational symmetry. Towards the left of the plot, the flowers are characterized by indistinguishable petals and small variations in symmetry. These trends, however, are not visually apparent from the embeddings of reflectional $\mathcal{T}\mathcal{I}$ curves (Sect. A.5).

**Fig. 7:**
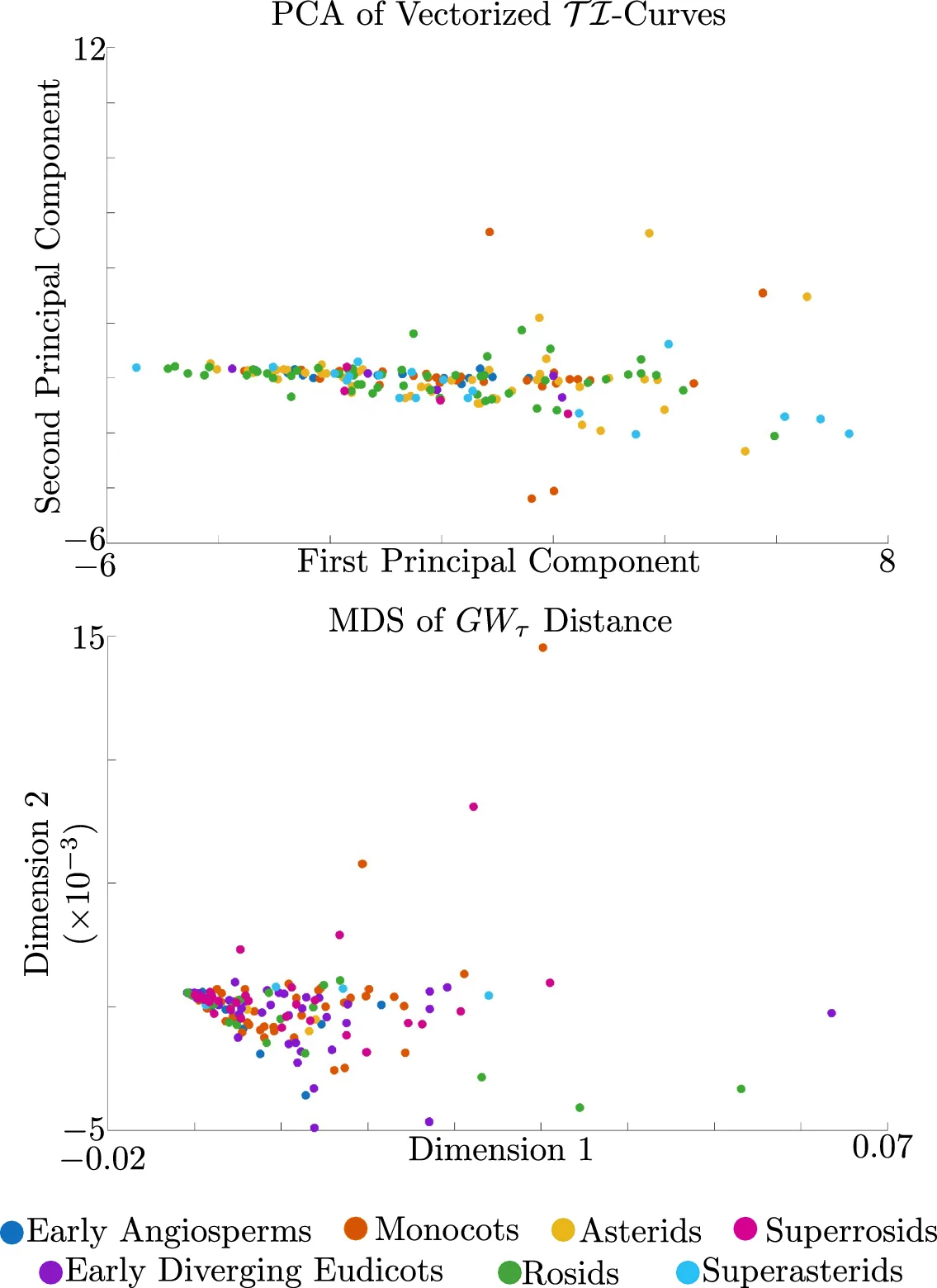
Alternative visualizations of symmetry landscapes. After vectorization, $\mathcal{T}\mathcal{I}$ curves are visualized using two approaches: PCA projection onto the first two principal components (top) and MDS embedding based on $GW_{\tau }$ distances (bottom). Both representations capture the overlap in symmetry types across clades and the continuous variations in symmetry that were present in the fPCA

The continuum of symmetry can be represented using a variety of visualization techniques. We choose fPCA here because it is a classical and well-established approach for comparing curves; however, there may be benefits to other visualizations depending on the application of interest. In Fig. 7, we present two such alternatives. Both methods begin by vectorizing the $\mathcal{T}\mathcal{I}$ curves as $(\mathcal{T}\mathcal{I}[\mu ,R_{\theta _1}],...,\mathcal{T}\mathcal{I}[\mu ,R_{\theta _N}])$ for some $0 \le \theta _1< ... < \theta _N \le 2\pi$. The top panel applies standard *principal component analysis* (PCA) to these vectors and projects them onto the first two principal components. The bottom panel computes the $GW_{\tau }$ distance (Kravtsova et al. 2023) between $\mathcal{T}\mathcal{I}$ vectors and embeds them in the plane using *multidimensional scaling* (MDS). $GW_{\tau }$ is an optimal transport metric derived from the Gromov-Wasserstein distance. $GW_{\tau }$ calculates the shape difference between two ordered data sets by representing data sets as metric-measure spaces. This measure consistently outperforms standard measures including Euclidean and Dynamic Time Warping (DTW) while being computationally efficient.

A systematic comparison of the strengths and limitations of these visualization approaches is left to future work.

### Fern Symmetries

The $\mathcal{T}\mathcal{I}$ framework accommodates various forms of symmetry. To demonstrate this, we analyzed the self-similarity (fractal symmetry) of simulated fern images under translation and scaling. Each simulated fern is the attractor of an iterated function system, where we vary a parameter to get different simulations (see Sect. A.6 for details).

**Fig. 8:**
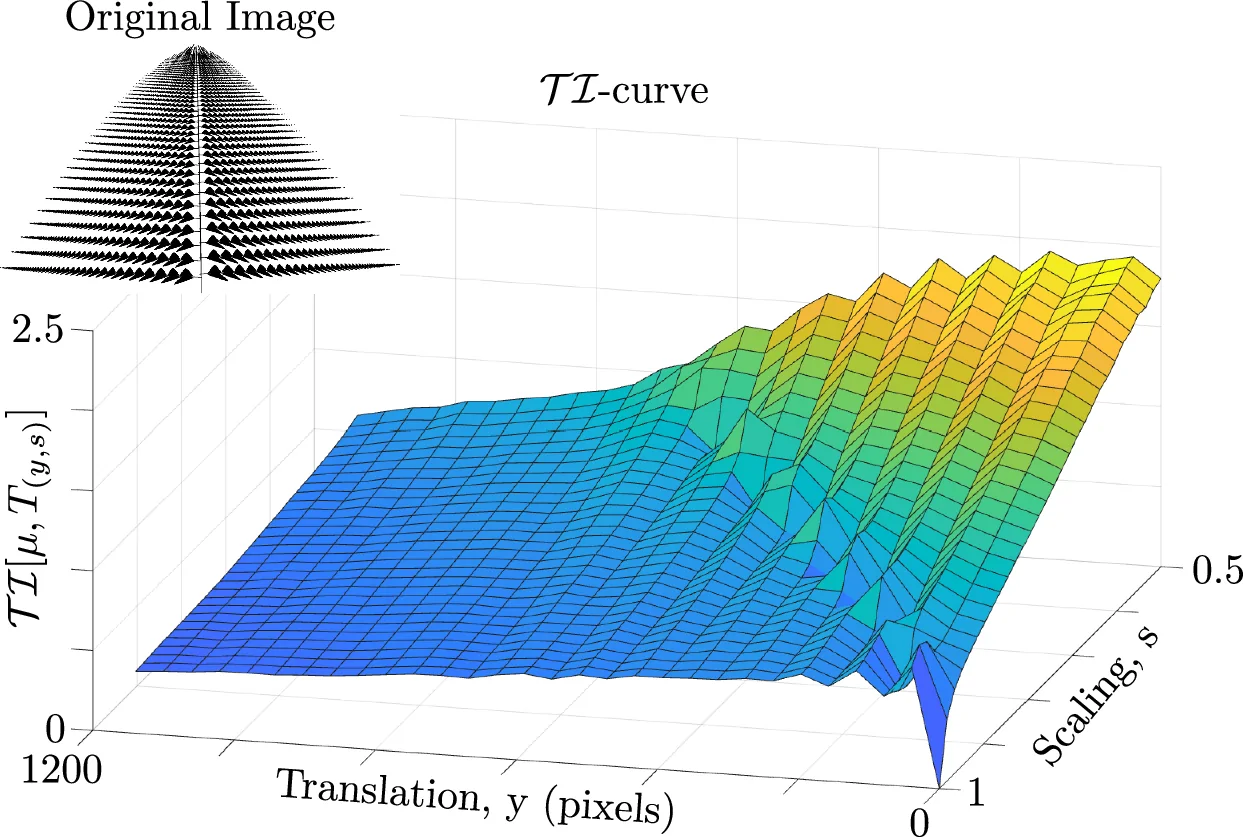
The $\mathcal{T}\mathcal{I}$ curve for the fern (top left) under vertical translation and scaling. Since the transformation $T_{(s,t)}$ relies on two parameters, the $\mathcal{T}\mathcal{I}$ curve is a 2-dimensional surface

The following set of transformations, expressed in homogeneous coordinates, represents the composition of scaling and translation along the positive *y*-axis:Given that the transformation relies on two parameters, the resulting $\mathcal{T}\mathcal{I}$ “curve” is a mapping from $\mathbb {R}^2\rightarrow \mathbb {R}$, as shown in Fig. 8.

**Fig. 9:**
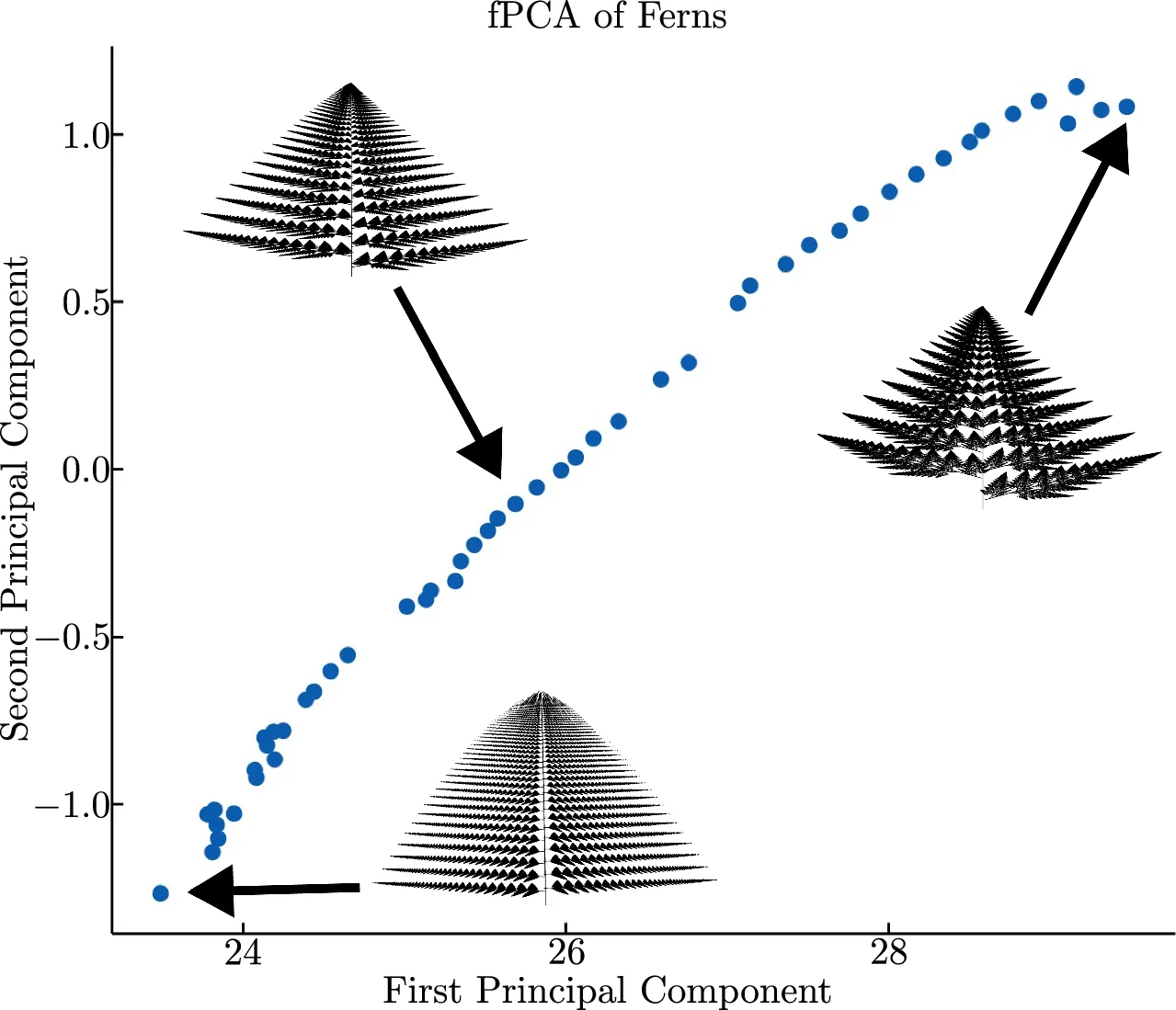
Functional principal component analysis of fern $\mathcal{T}\mathcal{I}$ curves. The first two principal components show a clear progression of symmetries, ranging from Barnsley-type ferns in the top right to *Cyclosorus* in the bottom left

**Fig. 11:**
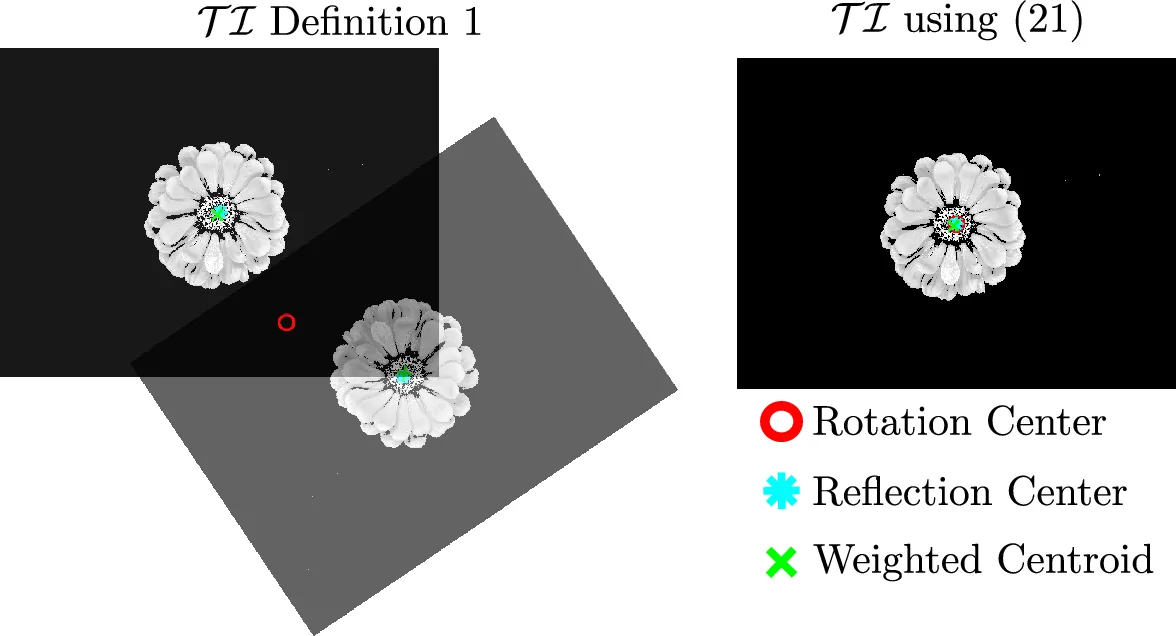
As discussed in Sect. A.1, using Equation (21) helps avoid artificial symmetries that may arise with Definition 1. For some images, this provides an improvement in the center detection. Left: the rotational center detected with Definition 1 is far from the flower. Rotating the image by 214π/180 about that center places the rotated flower entirely on the background of the original image, yielding an artificially small $\mathcal{T}\mathcal{I}$ value. Right: using Equation (21) for $\mathcal{T}\mathcal{I}$, the rotational center falls in the middle of the flower

We applied fPCA to these surfaces using the algorithm from Shi et al. (2022); the resulting projection onto the first two principal components is shown in Fig. 9. Complementing our observations in flowers, the ferns exhibit a symmetry continuum. There is a clear progression of symmetries, ranging from Barnsley-type ferns in the top right to *Cyclosorus*-type ferns in the bottom left, suggesting that future correlational studies should treat symmetry as a gradient rather than a discrete variable.

We note that the fPCA plot in Fig. 9 shows a linear, ordered curve. It is not obvious that this should be the case, i.e., that the linear family of IFS’s outlined in Sect. A.6 will trace out a linear curve in the space of $\mathcal{T}\mathcal{I}$ curves, since the map from any function μ to the associated $\mathcal{T}\mathcal{I}$ curve is nonlinear. We also may not expect a one-parameter family of IFS’s to trace a one-dimensional curve in the space of $\mathcal{T}\mathcal{I}$ curves, since the standard continuity argument does not necessarily apply here. In particular, despite the attractor $A_p$ of the IFS varying continuously in *p* with respect to the Hausdorff metric, the function $p \mapsto \chi _{A_p}$ (where $\chi _{A_p}$ is the indicator function of $A_p$) is not necessarily a continuous map into $L^1$, meaning we cannot directly compose the operations and apply Proposition 6. It is therefore an open question whether the map from the parameter *p* to the associated $\mathcal{T}\mathcal{I}$ curve is continuous in this case. We thus interpret the smooth linear progression in Fig. 9 as an empirical observation about this family of shapes rather than a consequence of the fern construction.

We also note that the set of fractals chosen here is relatively simple compared to real ferns, allowing us to analyze a two-parameter family of transformations to capture the fern symmetry. In more general settings, the analysis would require the exploration of symmetries in the full six-parameter family of affine transformations, which is possible with our approach, albeit computationally expensive.

## Discussion

We used $\mathcal{T}\mathcal{I}$ to represent symmetry as a broad, continuous spectrum of values and developed a user-friendly computational tool to measure and visualize the symmetries of image data. Applying this tool to angiosperm flower images recovered a spectrum of symmetries spanning evolutionarily-distinct clades. This revealed structure that traditional discrete classifications, such as radial versus bilateral symmetry, would have obscured and thus enabled biologically-meaningful conclusions about convergent evolution.

Traditional methods for measuring symmetry rely on user-selected landmarks. Our method still requires the user to select a family of symmetry transformations of interest and to ensure that the object is isolated against a uniform background. Despite this, the method replaces the difficult, subjective steps of landmark identification with more tractable and unbiased ones. No landmarks or homologous points are required, and the symmetry center, which would otherwise be a user-selected quantity, is detected automatically using computation (Definition 4). We demonstrate the fidelity of the method by investigating $\mathcal{T}\mathcal{I}$’s mathematical properties such as invariance, equivariance, and continuity. Equivariance and invariance ensure that the symmetry measure is agnostic to image orientation, while continuity ensures resilience to noise and measurement error. Together, these properties provide evidence that our method does not require image alignment or landmarking, and that the preprocessing steps required can be automated with standard tools (Sect. A.4).

Building on these theoretical guarantees, the computational pipeline measures and visualizes continuous symmetry across an image dataset: for each image, it locates the symmetry center, computes the $\mathcal{T}\mathcal{I}$ curve, and represents the symmetry as a two-dimensional data point via functional principal component analysis (fPCA). Representing symmetry as a function rather than a scalar or a categorical descriptor preserves more geometric information about each specimen, and the resulting “symmetry space” supports quantitative comparison across an entire dataset.

For example, in the fPCA plot of the floral images (Fig. 6), we observe diverse symmetry types, yet there is substantial overlap of symmetry types between clades. This overlap suggests that similar symmetries arose independently in different evolutionary lineages. While the overlap alone does not fully establish selective mechanisms, it provides strong evidence that similar symmetries may be favored in certain environmental conditions and motivates further investigation into the role of natural selection of symmetry. It would not be possible to see this evidence without a continuous measure of symmetry.

The $\mathcal{T}\mathcal{I}$ representation of symmetry also provides a foundation for testing correlations between symmetry and other quantitative traits. We anticipate that the continuous symmetry metric given by $\mathcal{T}\mathcal{I}$ will offer greater predictive power than traditional categorical classifications. The integration of $\mathcal{T}\mathcal{I}$ into predictive models has already shown promise in other contexts. For instance, Rostami et al. (2023) demonstrated that machine learning classifiers perform significantly better when incorporating information from $\mathcal{T}\mathcal{I}$. They demonstrated that “coarse-graining” types of actin networks based on shared symmetry led to much higher classification accuracy than classification based on the actin networks alone. In future work, we plan to use the framework proposed here to perform similar symmetry-informed classification in broader contexts. One promising application is to regular patterns of vegetation growth in dryland ecosystems  (see, e.g., Gandhi et al. (2019)), where $\mathcal{T}\mathcal{I}$ may help inform the classification of spatial patterns and quantify the deviation from spatial periodicity (Kästner et al. 2024).

Several limitations of this work also provide directions for future research. Our current computational framework is optimized for two-dimensional images with uniform backgrounds. Automated background removal techniques could improve the applications to general images. Furthermore, extending the framework to three-dimensional data, such as point clouds or Z-stacks (in confocal microscopy), will be necessary to capture the full complexity of many biological structures. More generally, the $\mathcal{T}\mathcal{I}$ framework can be applied beyond spatial symmetries, including higher-dimensional or nonphysical datasets where symmetry is not visually apparent yet still meaningful. Additionally, while we have established the descriptive strength of $\mathcal{T}\mathcal{I}$, the development of statistical models linking these curves to functional or mechanistic properties in biology remains an open challenge.

Such models would benefit from understanding how properties of a family of shapes are represented in the space of $\mathcal{T}\mathcal{I}$ curves. The fern family is instructive since the family of IFS’s has a linear parameterization in *p*, yet our framework does not predict that the fPCA plot of $\mathcal{T}\mathcal{I}$ curves would also be linear (as observed). Identifying conditions under which such properties of the symmetry can be inferred from the underlying model will provide a foundation for understanding how a model of a shape influences functional or mechanistic properties through symmetry.

Overall, our findings suggest that symmetry, which is traditionally treated as a qualitative, categorical trait, can be successfully transformed into a quantitative, continuous variable. By developing a high-throughput method that treats symmetry as a function rather than a scalar or categorical value, we enable better integration of symmetry into statistical and dynamical systems modeling and provide a more nuanced approach to investigating the prominent role of symmetry in biology.

## Supplementary Information

Below is the link to the electronic supplementary material.

Supplementary file 1 (pdf 92696 KB)

## Figures and Tables

**Fig. 10:**
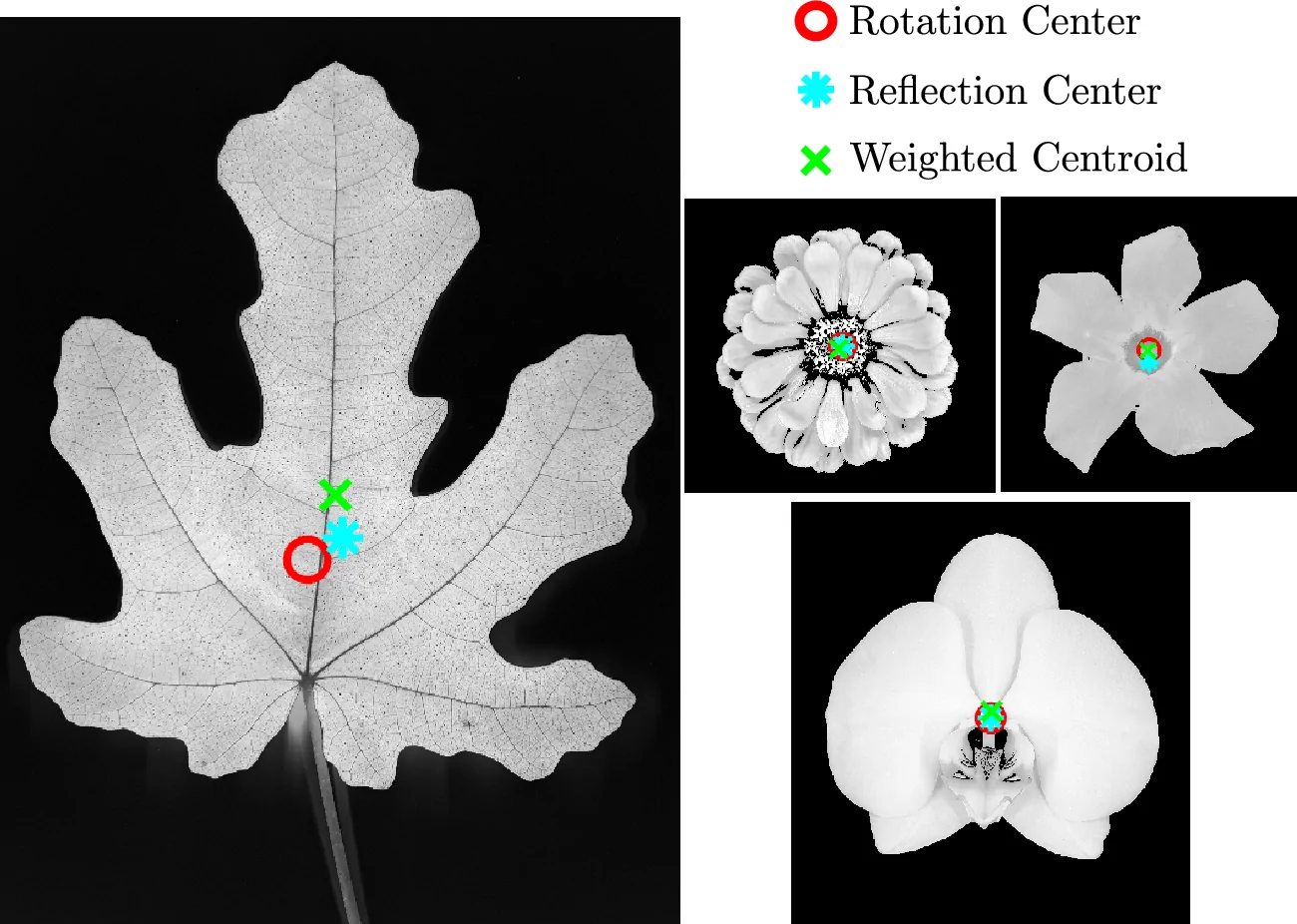
In four sample images, the symmetry center by rotation $\overline{x}^R$, reflection $\overline{x}^{\kappa }$, and the intensity-weighted centroid agree closely

**Fig. 12:**
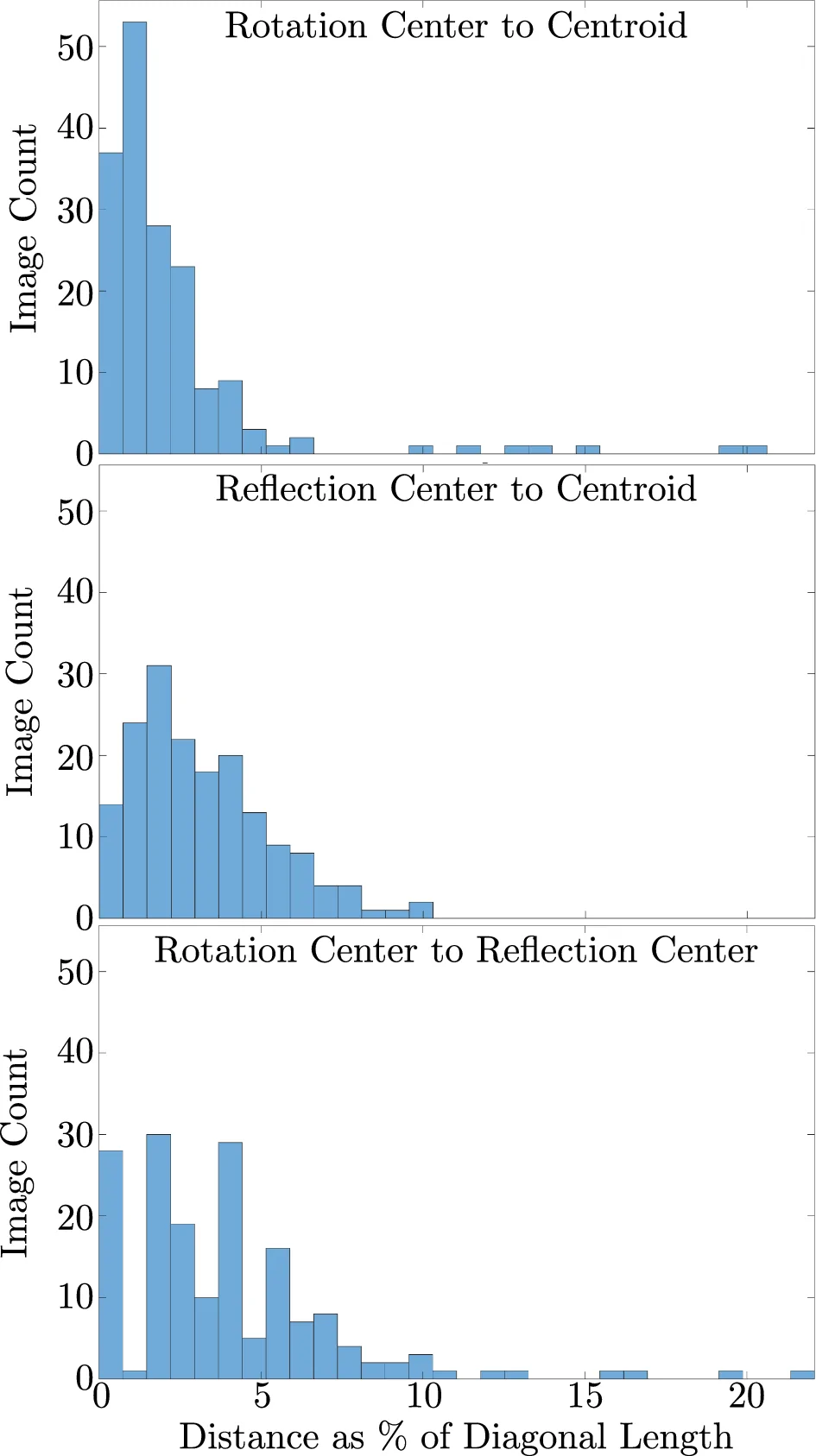
Across all flowers in the dataset from Byng et al. (2018), we compute the pairwise distances between the symmetry center by rotation, reflection, and the intensity-weighted centroid. The histograms shows the distance between each center as a percentage of the image’s diagonal length

**Fig. 13:**
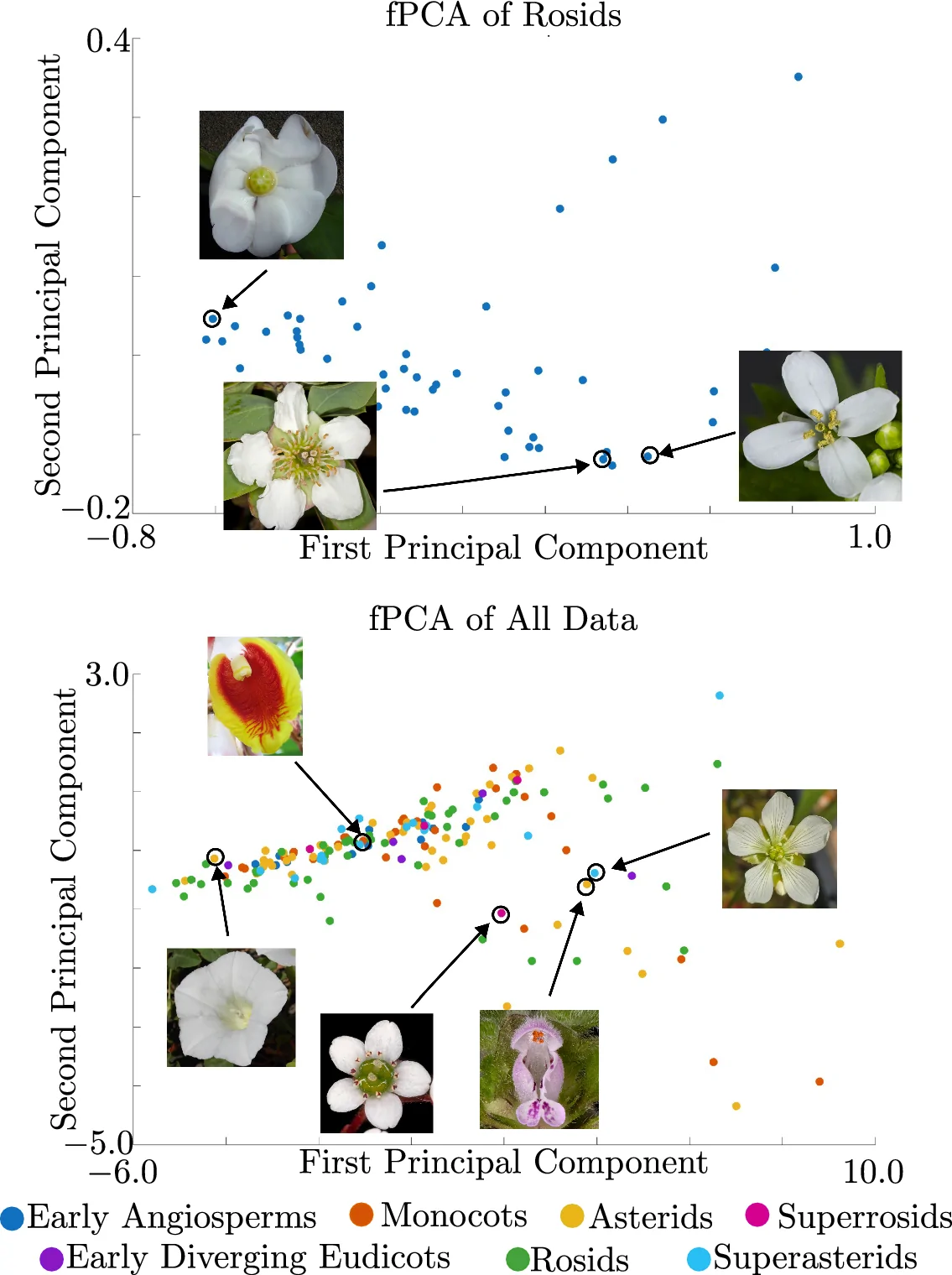
fPCA of $\mathcal{T}\mathcal{I}$ curves by reflection about the reflectional symmetry center ($\mathcal{T}\mathcal{I}[\mu _{\overline{x}^{\kappa }},\kappa _{\theta }])$ reveals a continuous landscape of symmetries, similar to the rotational $\mathcal{T}\mathcal{I}$ about the rotational symmetry center (Fig. 6). Top: rosid $\mathcal{T}\mathcal{I}$ curves projected onto the first two principal component symmetries, capturing 85.517% of the variance. Bottom: all projected $\mathcal{T}\mathcal{I}$ curves, color-coded by clade, capturing 85.538% of the variance. The flower images presented on the graphs are stock images that closely match the image data used for our analysis (Descouens 2022; Game 2017; Starr and Starr 2026; North Queensland Plants 2026; Reese 2024; falcon_311 2025; Carr 2005; Edison and Ford Winter Estates 2010; Smith 2024). Clades remain intermixed as in Fig. 6; however, visual separation into 4-fold and 5-fold symmetry is not apparent in the rosids, and separation into bilateral and rotational symmetry is not apparent in the full dataset

**Algorithm 1:**
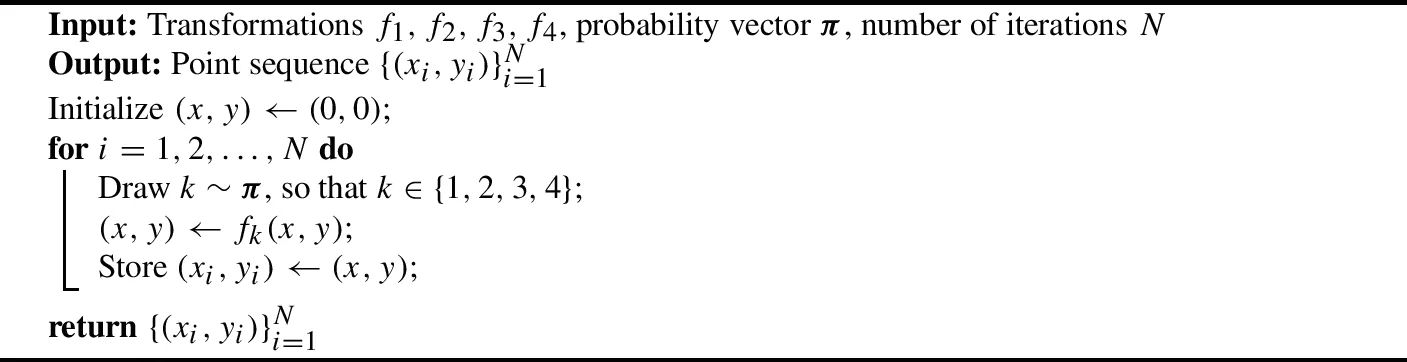
Iterated Function System (IFS) Attractor

## Data Availability

The code referenced in this paper is available at GitHub (2025). It contains the computational pipeline used to create all $\mathcal{T}\mathcal{I}$ curves and fPCA plots presented. All figures were created using MATLAB and Inkscape.

## Appendix

### Alternative Definitions of $\mathbf {\mathcal{T}\mathcal{I}}$

In addition to the hypotheses in Definition 1 for transformation information, Ciocanel et al. (2025) require $\mu : D \rightarrow \mathbb {R}_{>0}$ to be normalized. That is, it must satisfy $\int _D \mu (x) \ dx = 1$. From the definition of $\mathcal{T}\mathcal{I}$, we can easily see that normalizing μ amounts to scaling the $\mathcal{T}\mathcal{I}$ curve. Indeed, if a distribution $\overline{\mu }$ is normalized by a scaling factor $s\in \mathbb {R}$ so that $\mu := s\overline{\mu }$, then for any transformation $A\in \text {Aff}(n)$,

$$\begin{aligned} \mathcal{T}\mathcal{I}[\mu ,A]&= \mathcal{T}\mathcal{I}[s\overline{\mu },A] \\&= \int _{\widetilde{D}} s\overline{\mu } \log \left( \frac{s\overline{\mu }}{A(s\overline{\mu })}\right) \\&= s\int _{\widetilde{D}} \overline{\mu } \log \left( \frac{s\overline{\mu }}{sA\overline{\mu }}\right) \\&= s\int _{\widetilde{D}} \overline{\mu } \log \left( \frac{\overline{\mu }}{A\overline{\mu }}\right) \\&= s\mathcal{T}\mathcal{I}[\overline{\mu },A]. \end{aligned}$$

The decision to use normalization thus depends on the analytical goal. We recommend applying normalization if the objective is to compare an object’s shape independent of its size. Conversely, normalization should be omitted when size disparity between objects is a meaningful component of the data analysis, particularly when comparing $\mathcal{T}\mathcal{I}$ curves using data visualization methods that compare $\mathcal{T}\mathcal{I}$ curves componentwise. It is important to note, however, that the approximate symmetries and maximal asymmetries are identical regardless of whether normalization is applied.

A potential issue with the $\mathcal{T}\mathcal{I}$ definition is that integrating over the domain $\widetilde{D}$ can “cut off” parts of the transformed function Aμ for some $A\in \text {Aff}(n)$, creating artificial symmetry (see Fig. 3). We avoid this issue by embedding the objects of interest within a sufficiently large, uniform background (Proposition 1). An alternative approach is to extend $\overline{\mu }: D \subset \mathbb {R}^n \rightarrow \mathbb {R}_{>0}$ to a function μ defined on all of $\mathbb {R}^n$. Assume that $\overline{\mu }(x)>\delta$ for some δ>0 for all x∈D. Then let:

21 $$\begin{aligned} \mu (x)= {\left\{ \begin{array}{ll} \overline{\mu }(x) & x\in D \\ \delta & x\in \mathbb {R}^n \setminus D. \end{array}\right. } \end{aligned}$$

With this definition, $\widetilde{D} = \mathbb {R}^n$ for any transformation $A\in \text {Aff}(n)$, and thus $\mathcal{T}\mathcal{I}[\mu ,A]$ is calculated by integrating over $\mathbb {R}^n$.

### Additional Mathematical Properties of $\mathcal{T}\mathcal{I}$

#### Proof of equation (15)

$$\begin{aligned} F_{\text {rot}}(R_{\varphi }\mu )&= \int (\mu \circ R_{\varphi }^{-1}) \log \left( \frac{\mu \circ R_{\varphi }^{-1}}{\mu \circ {R_{\varphi }^{-1}}\circ R_{\theta }^{-1}}\right) \\&= \int \mu \log \left( \frac{\mu }{\mu \circ {R_{\varphi }^{-1}}\circ R_{\theta }^{-1}\circ R_{\varphi }}\right) &  (x\mapsto R_{\varphi }x) \\&= \int \mu \log \left( \frac{\mu }{\mu \circ R_{\theta }^{-1}}\right) &  \text {(rotations commute)} \\&= \mathcal{T}\mathcal{I}_{\text {rot}}(\theta ) \\&=F_{rot}(\mu ). \ \end{aligned}$$

□

#### Proof of equation (16)

$$\begin{aligned} F_{\text {rot}}(\kappa _{\varphi }\mu )&= \int (\mu \circ \kappa _{\varphi }^{-1})\log \left( \frac{\mu \circ \kappa _{\varphi }^{-1}}{\mu \circ \kappa _{\varphi }^{-1}\circ R_{\theta }^{-1}}\right) \\&=\int \mu \log \left( \frac{\mu }{\mu \circ \kappa _{\varphi }^{-1}\circ R_{\theta }^{-1} \circ \kappa _{\varphi }}\right) . &  (x\mapsto \kappa _{\varphi }x) \end{aligned}$$

Now, we will show that $\kappa _{\varphi }^{-1}\circ R_{\theta }^{-1} \circ \kappa _{\varphi }=R_{-\theta }^{-1}$. Taking $z=(x,y) \in \mathbb {C}$, rotations $R_{\theta }$ and reflections $\kappa _{\theta }$ on $\mathbb {R}^2$ are equivalent to the following transformations on $\mathbb {C}$

22 $$\begin{aligned} R_{\theta } z&= e^{i\theta }z \end{aligned}$$

23 $$\begin{aligned} \kappa _{\theta }z&=e^{2i\theta }\overline{z}. \end{aligned}$$

Furthermore, it is easy to see that $\kappa _{\theta }^{-1} = \kappa _{\theta }$ and $R_{\theta }^{-1} = R_{-\theta }$. Therefore,

$$\begin{aligned} \kappa _{\varphi }^{-1}\circ R_{\theta }^{-1} \circ \kappa _{\varphi }z&= \kappa _{\varphi }^{-1} \circ R_{\theta }^{-1} e^{2i\varphi }\overline{z} \\&= \kappa _{\varphi }^{-1}\circ e^{-i\theta } e^{2i\varphi }\overline{z} \\&= e^{2i\varphi }e^{i\theta }e^{-2i\varphi } z \\&= e^{i\theta } z \\&= R_{\theta }z \\&=R_{-\theta }^{-1}z. \end{aligned}$$

So,

$$\begin{aligned} F_{\text {rot}}(\kappa _{\varphi }\mu )&= \int \mu \log \left( \frac{\mu }{\mu \circ \kappa _{\varphi }^{-1}\circ R_{\theta }^{-1} \kappa _{\theta }}\right) \\&= \int \mu \log \left( \frac{\mu }{\mu \circ R_{-\theta }^{-1}}\right) \\&= \mathcal{T}\mathcal{I}_{\text {rot}}(-\theta ) \\&=\overline{\kappa }_0F_{\text {rot}}(\mu ). \end{aligned}$$

□

#### Proof of equation (17)

$$\begin{aligned} F_{\text {ref}}(R_{\varphi }\mu )&= \int (\mu \circ R_{\varphi }^{-1}) \log \left( \frac{\mu \circ R_{\varphi }^{-1}}{\mu \circ {R_{\varphi }^{-1}}\circ \kappa _{\theta }^{-1}}\right) \\&= \int \mu \log \left( \frac{\mu }{\mu \circ {R_{\varphi }^{-1}}\circ \kappa _{\theta }^{-1}\circ R_{\varphi }}\right) . &  (x\mapsto R_{\varphi } x) \end{aligned}$$

Using complex coordinates,

$$\begin{aligned} R_{\varphi }^{-1}\circ \kappa _{\theta }^{-1}\circ R_{\varphi }z&=R_{\varphi }^{-1}\circ \kappa _{\theta }^{-1}e^{i\varphi }z \\&= R_{\varphi }^{-1} e^{2i\theta }e^{-i\varphi } \overline{z} \\&= e^{-i\varphi } e^{2i\theta }e^{-i\varphi } \overline{z} \\&= e^{2i(\theta -\varphi )}\overline{z}\\&= \kappa _{\theta -\varphi }^{-1} z. \end{aligned}$$

Thus,

$$\begin{aligned} F_{\text {ref}}(R_{\varphi }\mu )&= \int \mu \log \left( \frac{\mu }{\mu \circ {R_{\varphi }^{-1}}\circ \kappa _{\theta }^{-1}\circ R_{\varphi }}\right) \\&=\int \mu \log \left( \frac{\mu }{\mu \circ \kappa _{\theta -\varphi }^{-1}}\right) \\&=\mathcal{T}\mathcal{I}_{\text {ref}}(\theta -\varphi ) \\&=\mathcal{T}\mathcal{I}_{\text {ref}}(\overline{R}_{\varphi }^{-1}(\theta )) \\&= \overline{R}_{\varphi }F_{\text {ref}}(\mu ). \end{aligned}$$

□

#### Proof of equation (18)

$$\begin{aligned} F_{\text {ref}}(\kappa _{\varphi }\mu )&= \int (\mu \circ \kappa _{\varphi }^{-1}) \log \left( \frac{\mu \circ \kappa _{\varphi }^{-1}}{\mu \circ {\kappa _{\varphi }^{-1}}\circ \kappa _{\theta }^{-1}}\right) \\&= \int \mu \log \left( \frac{\mu }{\mu \circ {\kappa _{\varphi }^{-1}}\circ \kappa _{\theta }^{-1}\circ \kappa _{\varphi }}\right) &  (x\mapsto \kappa _{\varphi }x). \end{aligned}$$

Using complex coordinates,

$$\begin{aligned} \kappa _{\varphi }^{-1}\circ \kappa _{\theta }^{-1}\circ \kappa _{\varphi }z \\&= \kappa _{\varphi }^{-1}\kappa _{\theta }^{-1}(e^{2i\varphi }\overline{z}) \\&= \kappa _{\varphi }^{-1}(e^{2i(\theta -\varphi )}z) \\&=e^{2i(2\varphi - \theta )}\overline{z} \\&=\kappa _{2\varphi - \theta }z, \end{aligned}$$

so

$$\begin{aligned} F_{\text {ref}}(\kappa _{\varphi }\mu )&= \mathcal{T}\mathcal{I}_{\text {ref}}(2\varphi - \theta ) \\&= \mathcal{T}\mathcal{I}_{\text {ref}}(\overline{\kappa }_{\varphi }^{-1} (\theta )) \\&= \kappa _{\varphi } F_{\text {ref}}(\mu ). \end{aligned}$$

□

#### Proposition 6

($\mathbf {\mathcal{T}\mathcal{I}}$ is Lipshitz continuous) Let let $\{T_a\}_{a\in \mathcal {A}} \subset \text {Aff}(n)$ be such that

$$\begin{aligned} \text {sup}_{a\in \mathcal {A}}\{|\det (T_a)|\} < \infty , \end{aligned}$$

fix $D \subset \mathbb {R}^n$ such that |D|<∞ (Lebesgue measure of *D*), and define

$$\begin{aligned} \mathcal {P}_{\delta ,M}&= \{ f \in L^1(D) : 0<\delta< f(x) < M \ a.e. \} \\ \mathcal {Y}&=\{ f: \mathcal {A} \rightarrow \mathbb {R}\} \end{aligned}$$

where $\mathcal {P}_{\delta ,M}$ has the metric induced by the $L^1$ norm, and $\mathcal {Y}$ has the sup norm. Lastly, define $F: \mathcal {P}_{\delta ,M} \rightarrow \mathcal {Y}$ as $F: \mu \mapsto \mathcal{T}\mathcal{I}(a)$ where $\mathcal{T}\mathcal{I}(a)$ is the $\mathcal{T}\mathcal{I}$ curve of $\mathcal{T}\mathcal{I}[\mu ,T_a]$ as a function of $\mathcal {A}$. Then *F* is Lipshitz continuous, that is:

$$\begin{aligned} |F(\mu ) - F(\eta )|_{\text {sup}(a)} \le L \Vert \mu -\eta \Vert _{L^1(D)} \end{aligned}$$

for some constant *L*.

#### Proof

Take some arbitrary $T_a$, and let $\mu ,\eta \in \mathcal {P}_{\delta , M}$. We can write

$$\begin{aligned} \mathcal{T}\mathcal{I}[\mu ,T_a]=\int _{\widetilde{D}} \Phi (\mu ,T_a\mu ) \end{aligned}$$

where

$$\begin{aligned} \Phi (u,v) = u\log \left( \frac{u}{v}\right) \,. \end{aligned}$$

We will first show that the function $\Phi (u,v) : \mathbb {R}^2 \rightarrow \mathbb {R}$ is Lipshitz continuous on $S:=(\delta ,M) \times (\delta ,M)$. Indeed, the partial derivatives

24 $$\begin{aligned} \Phi _u&= \log \left( \frac{u}{v}\right) + 1 \end{aligned}$$

25 $$\begin{aligned} \Phi _v&=-\frac{u}{v} \end{aligned}$$

are bounded on the convex set *S*. By the mean value theorem. For any $\left(u,v\right),\left(u^{\prime },v^{\prime }\right)\in S$, there exists a point c∈S such that

26 $$\begin{aligned} \Phi (u,v)-\Phi (u',v')&= [(u,v)-(u',v')] \cdot \nabla \Phi (c) \end{aligned}$$

27 $$\begin{aligned}&= \Phi _u(c) (u-u') + \Phi _v(c)(v-v') \end{aligned}$$

where · denotes the standard Euclidean inner product (or dot product), and $\nabla \Phi (c)$ denotes the gradient of Φ evaluated at *c*. Let

$$\begin{aligned} K_1&= \sup _{c\in S} |\Phi _u(c)|\\ K_2&= \sup _{c\in S} |\Phi _v(c)|, \end{aligned}$$

and take $K = \text {max}\{K_1,K_2\}$. Now, taking the absolute value on both sides of (26), (27), we have

28 $$\begin{aligned}&|\Phi (u,v)-\Phi (u',v')| \le K (|u-u'| + |v-v'|) \end{aligned}$$

Therefore, Φ is Lipshitz when considering the domain $\mathbb {R}^2$ with the *Manhattan norm*.

We can then write

$$\begin{aligned}&|\mathcal{T}\mathcal{I}[\mu , T_a] - \mathcal{T}\mathcal{I}[\eta ,T_a]| = \Bigg |\int _{\widetilde{D}} \Phi (\mu ,T_a\mu ) - \int \Phi (\eta ,T_a\eta )\Bigg | \\&= \Bigg |\int _{\widetilde{D}} \Phi (\mu ,T_a\mu ) - \Phi (\eta ,T_a\eta )\Bigg | \\&\le \int _{\widetilde{D}} |\Phi (\mu ,T_a\mu ) - \Phi (\eta ,T_a\eta )| \\&\le K \int _{\widetilde{D}} (|\mu -\eta | + |T_a\mu - T_a\eta |) &  \text {equation (28)} \\&= K \left( \int _{\widetilde{D}} |\mu -\eta | + \int _{\widetilde{D}} |\mu \circ T_a^{-1} - \eta \circ T_a^{-1}|\right) \\&\le K(1+|\det (T_a)|)\int _{\widetilde{D}} |\mu -\eta | \\&\le K(1+|\det (T_a)|)\Vert \mu -\eta \Vert _{L^1(D)} \end{aligned}$$

Let $L:=K(1+\text {sup}_{a\in \mathcal {A}}\{| \det (T_a)|\})$, where the supremum is finite by hypothesis. Then for every *a*,

$$\begin{aligned} |\mathcal{T}\mathcal{I}[\mu , T_a] - \mathcal{T}\mathcal{I}[\eta ,T_a]| \le L \Vert \mu -\eta \Vert _{L^1(D)}, \end{aligned}$$

or equivalently

$$\begin{aligned} |F(\mu )-F(\eta )|_{\text {sup}(a)} \le L\Vert \mu -\eta \Vert _{L^1(D)}. \ \end{aligned}$$

□

#### Proposition 7

(Conditions for equivalent reflections and rotations) Let $\mu :\mathbb {R}^2 \rightarrow \mathbb {R}$. If there exists some $\varphi \in [0,2\pi )$ such that $\kappa _{\varphi }\mu =\mu$, then for all $\theta \in [0,2\pi )$,

$$\begin{aligned} \kappa _{\theta }\mu = R_{2(\theta -\varphi )}\mu . \end{aligned}$$

#### Proof

As in (22), (23), we will use the fact that $\mathbb {R} \cong \mathbb {C}$ (i.e. they are isomorphic as vector spaces). Assume that there exists some $\varphi \in [0,2\pi )$ such that $\kappa _{\varphi }\mu \equiv \mu$. Then for any $z\in \mathbb {C}$

$$\begin{aligned} \kappa _{\theta }\mu (z)&= \mu (e^{2i\theta }\overline{z}) \\&=\kappa _{\varphi }\mu (e^{2i\theta }\overline{z}) &  \text {(by assumption)} \\&=\mu (e^{2i\varphi }e^{-2i\theta } z) \\&=\mu (e^{2i(\varphi -\theta )}z) \\&= \mu (R_{2(\varphi -\theta )}z) \\&= R_{2(\theta -\varphi )}\mu (z). \end{aligned}$$

□

### Comparing Approaches for Center Detection

Definition 4 locates the symmetry center by maximizing the observed symmetry over candidate points, which is considerably more computationally expensive than computing the intensity-weighted centroid (4). Here we compare the rotational and reflectional symmetry centers to the intensity-weighted centroid, and to each other, across images in the floral dataset. All distances are reported as a percentage of the image’s diagonal length. When computing the symmetry centers, we use the definition of $\mathcal{T}\mathcal{I}$ given by (21) to avoid artificial symmetries and observe improvements as shown in Fig. 11. Rather than integrating over $\widetilde{D}$, the intersection of the domains of the original and transformed image, we instead integrate over the union of the images’ domains, trivially extending each image into the domain where it is not defined by setting the pixel intensity to a preselected small value δ. For more details on symmetry center detection, see Sect. 4.4 (Fig. 10).

Figure 12 shows the distribution of distances across the floral dataset for all three centers. Table 1 provides summary statistics for each distribution. The rotational symmetry center $\overline{x}^R$ agrees most closely with the centroid, with a median displacement of 1.43% and 93.6% of images below 5% displacement. The reflectional center $\overline{x}^{\kappa }$ is displaced somewhat further from the centroid (median 2.75%) but with a tighter distribution as the distance is less than 10% in all images. The two symmetry centers differ from one another more than either differs from the centroid (median 3.57%).

Despite close agreement of the centers across most of the dataset, in 3.5% of images (6 of 171) the distance between $\overline{x}^R$ and the centroid is over 10%, and in 4.7% of images (8 of 171) the distance between symmetry centers is more that 10% apart. These cases show that there are cases where the choice of center selection method, and hence the choice among the four $\mathcal{T}\mathcal{I}$ curves of Sect. 4, may be significant. Further investigation is required to understand why the centers differ in some images but not in others.

### Details of Preprocessing

If a grayscale image is represented by the matrix $M=(m_{ij})$, its negative is simply given by $N = (255-m_{ij})$. As before, we define the representative function of the image as

$$\begin{aligned} \overline{\mu }(i,j)=(255-m_{ij})+\delta \,, \end{aligned}$$

where δ>0 is a small value added to avoid zero pixel values, thus ensuring $\mathcal{T}\mathcal{I}$ is defined (see Fig. S4).

In cases where the background of an image has significant variations in light intensity and color, the image requires additional preprocessing so that the $\mathcal{T}\mathcal{I}$ measure does not detect properties of the background. If every point in the background has lower light intensity than the points in the object, then we can use thresholding to remove the background noise. Mathematically, consider a grayscale image represented by the intensity matrix $M=(m_{ij})$, and suppose the object of interest is represented with a set of pixels F⊂D. If for every (i,j)∈F and $(\ell ,k)\in D\setminus F$, we have

29 $$\begin{aligned} m_{\ell k} < m_{ij}, \end{aligned}$$

then we can choose a threshold $T:= \min _{(i,j)\in F} \{m_{ij}\}$ and set

30 $$\begin{aligned} \overline{\mu }(i,j)= {\left\{ \begin{array}{ll} 1 & m_{ij} < T \\ m_{ij} & m_{ij} \ge T. \end{array}\right. } \end{aligned}$$

Then $\overline{\mu }$ retains the grayscale image’s pixel intensity on the object *F* and achieves the minimal pixel intensity on the complement D\F. Alternatively, if we wish to investigate symmetry in the object’s global boundary geometry rather than in textures or internal surface features, we can consider the binarized image

31 $$\begin{aligned} \overline{\mu }(i,j)= {\left\{ \begin{array}{ll} 1 & m_{ij} < T \\ 255 & m_{ij} \ge T. \end{array}\right. } \end{aligned}$$

The difference between thresholding (30) and binarization (31) can be observed in Fig. S2.

In general, the threshold *T* can be difficult to find and varies between images within a dataset. Fortunately, there are well-established methods for automated preprocessing. In our computational pipeline (GitHub 2025), we use Otsu’s method (available via the MATLAB function otsuthresh), which finds a threshold *T* that maximizes the variance in pixel intensity between the groups created by the threshold.

Sometimes, thresholding is insufficient to distinguish between the background and the object since (29) is not satisfied. To resolve this issue, we propose taking the largest connected component of the binarized image or filtering an image before thresholding. To take the largest connected component, one can use the MATLAB function |bwareafilt|. Alternatively, filters smooth an image by averaging pixel values over a neighborhood. For example, we can apply a Gaussian filter with the MATLAB function imgaussfilt, which makes subsequent thresholding more effective. In fig. S4, when the standard deviation of the Gaussian filter is σ=0 (i.e. no change to the image), there are light pixels near the bottom of the leaf, which could bias the $\mathcal{T}\mathcal{I}$ curve. Increasing the standard deviation “smooths out” the image and removes background noise.

**Table 1:** Summary statistics of the pairwise distances between the rotational symmetry center $\overline{x}^{R}$, the reflectional symmetry center $\overline{x}^{\kappa }$, and the intensity-weighted centroid, across 171 floral images. Distances are expressed as a percentage of image diagonal length

Pair	Mean	Median	$p_{90}$	<5%	<10%
$\overline{x}^{R}$ to centroid	2.24	1.43	3.98	93.6%	96.5%
$\overline{x}^{\kappa }$ to centroid	3.18	2.75	6.09	81.9%	100%
$\overline{x}^{R}$ to $\overline{x}^{\kappa }$	3.91	3.57	7.41	69.6%	95.3%

### Reflectional $\mathcal{T}\mathcal{I}$ for Floral Dataset

The analysis in Sect. 5.1 considers $\mathcal{T}\mathcal{I}$ by rotation about the rotational symmetry center. Here we present the corresponding analysis for the $\mathcal{T}\mathcal{I}$ curve by reflection about the reflectional symmetry center ($\mathcal{T}\mathcal{I}[\overline{\mu }_{\overline{x}^{\kappa }},\kappa _{\theta }]$, Sect. 4.4), with fPCA plots for the $\mathcal{T}\mathcal{I}$ curves shown in Fig. 13. Note that the reflectional symmetry centers were computed taking the original definition of $\mathcal{T}\mathcal{I}$ rather than integrating over the union the original and transformed images’ domains as described by (21) and in Sect. A.3.

The two analyses are not independent. By Proposition 7, an object with exact reflection symmetry has rotational and reflectional $\mathcal{T}\mathcal{I}$ curves that determine each other, so in a dataset of exactly mirror-symmetric flowers, the $\mathcal{T}\mathcal{I}$ curves for rotation and reflection would contain the same information. Real flowers deviate from exact mirror symmetry, and the difference can be seen when comparing Fig. 6 and Fig. 13. We hypothesize that part of the difference is also because rotational $\mathcal{T}\mathcal{I}$ is orientation-agnostic while reflectional $\mathcal{T}\mathcal{I}$ is not (Proposition 5).

When analyzing the fPCA plots for the reflectional $\mathcal{T}\mathcal{I}$, the qualitative conclusions of Sect. 5 are unchanged. First, symmetry appears as a continuum rather than being grouped into distinct categories. Second, the $\mathcal{T}\mathcal{I}$ curves in the fPCA plot of all flowers (Fig. 13, bottom) overlap across distinct clades, suggesting convergent evolution of floral symmetry. However, the structure that was visually interpretable in the rotational analysis is less apparent here. In particular, the rosid fPCA plot does not separate into branches corresponding to the 4-fold and 5-fold rotational symmetry, and the full fPCA plot does not show separation into bilateral and radial symmetry. Thus, $\mathcal{T}\mathcal{I}$ by reflection is capturing information that may not be accessible via conventional approaches, like visual inspection of the first two components in PCA.

### Details of Fern Simulations

To obtain simulated images of ferns, we numerically compute the attractor of the following iterated function system (IFS) (Barnsley 1993), where $p\in \mathbb {R}$ is a parameter.

$$\begin{aligned} f_1(x,y)&= \begin{pmatrix} 0 & 0 \\ 0 & 0.16 \end{pmatrix} \begin{pmatrix} x \\ y \end{pmatrix} + \begin{pmatrix} 0 \\ -0.4p\end{pmatrix}\\ f_2(x,y)&= \begin{pmatrix} 0.85(1-p)+0.95p & 0 \\ -0.04(1-p)-0.005p & 0.85(1-p)+0.93p \end{pmatrix} \begin{pmatrix} x \\ y \end{pmatrix} + \begin{pmatrix} -0.002p \\ 1.6(1-p)+0.5p \end{pmatrix} \\ f_3(x,y)&= \begin{pmatrix} 0.2(1-p)+0.035p & -0.26(1-p)-0.2p \\ 0.23(1-p)+0.16p & 0.22(1-p)+0.04p \end{pmatrix} \begin{pmatrix} x \\ y \end{pmatrix} + \begin{pmatrix} -0.09p \\ 1.6(1-p)+0.02p \end{pmatrix} \\ f_4(x,y)&= \begin{pmatrix} -0.15(1-p)-0.04p & 0.28(1-p)+0.2p \\ 0.26(1-p)+0.16p & 0.24(1-p)+0.04p \end{pmatrix} \begin{pmatrix} x \\ y \end{pmatrix} + \begin{pmatrix} 0.083p \\ 0.44(1-p)+0.12p \end{pmatrix} \end{aligned}$$

When p=0, the attractor is a Barnsley-type fern as shown in Fig. 9 (top right) and when p=1, the attractor is a *Cyclosorus*-type fern as in Fig. 9 (bottom left).

In our analysis, we include 51 images, which are given by numerically computing the attractor of the IFS with $p=0, \ 0.02, \ 0.04, \ ..., \ 1.00$.

To simulate an attractor, we fix *p* and consider a random walk given by the IFS. Specifically, we start at (x,y)=(0,0). We select one of the transformations $f_1,f_2,f_3,f_4$ based on the probability distribution $\boldsymbol{\pi }$ given by

$$\begin{aligned} P(f_1 \text { is selected})&=0.01(1-p)+0.02p \\ P(f_2 \text { is selected})&=0.85(1-p)+0.84p \\ P(f_3 \text { is selected})&=0.07 \\ P(f_4 \text { is selected})&=0.07. \end{aligned}$$

Once $f_i$ is selected, we update (*x*, *y*) by applying $f_i$ and repeat the process. This is made explicit with the following pseudocode.

We consider a scatter plots of the points $\{(x_i,y_i)\}_{i=1}^N$ and use the MATLAB function exportgraphics to convert the scatter plot to an image file.
