# Supplementary material for: A High Throughput and User-Friendly Computational Tool for Quantifying and Visualizing Symmetry Variation in Image Datasets
Source: Bull Math Biol. 2026 Oct 7;88(11):198. doi: 10.1007/s11538-026-01754-9 (PMC13645982; doi:10.1007/s11538-026-01754-9)
Supplement: Supplementary file 1 [file 11538_2026_1754_MOESM1_ESM.pdf]

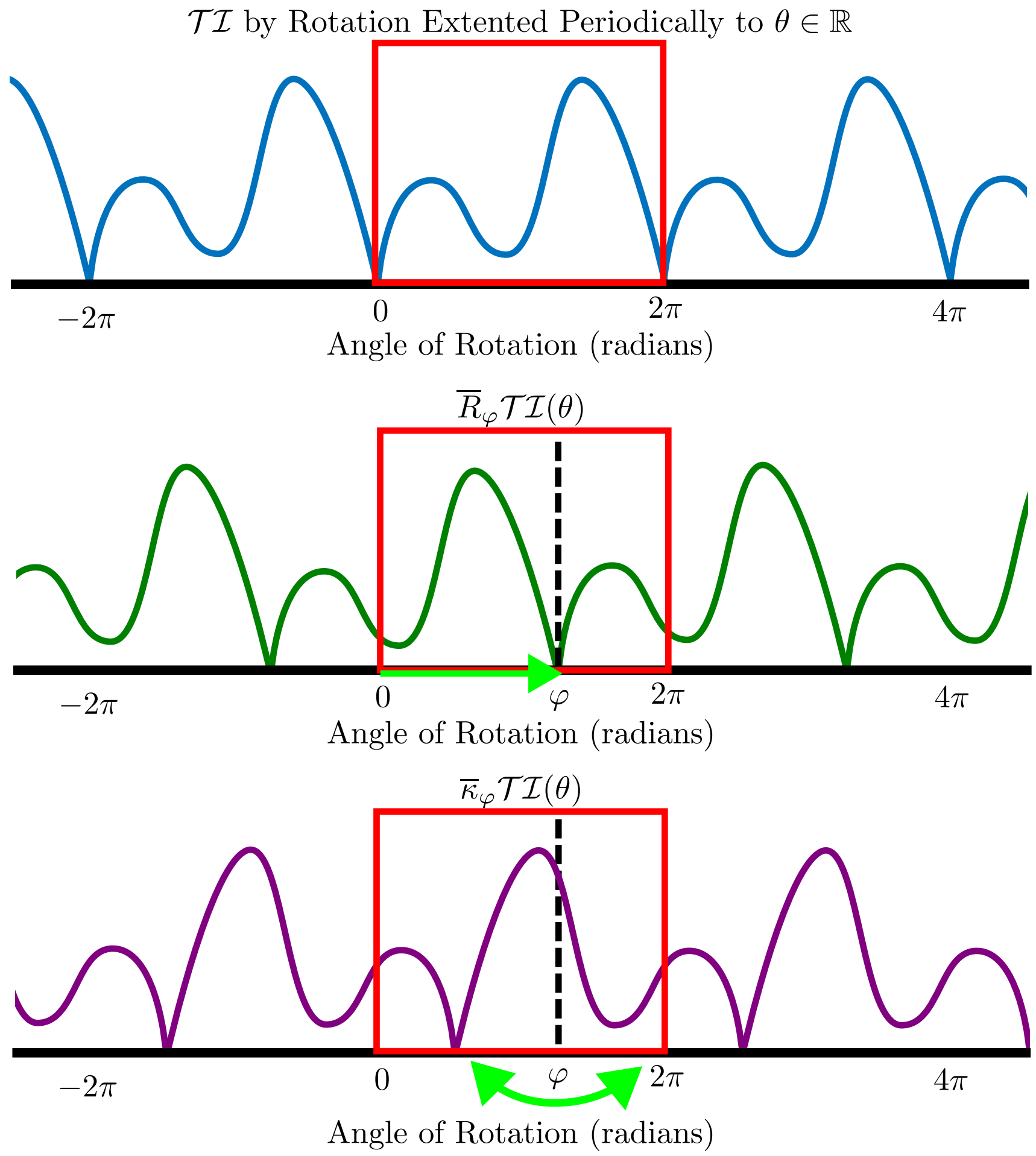

Figure 1: We show how $\mathcal{TI}$ is transformed under the actions of $\overline{R}_\varphi$ and $\overline{\kappa}_\varphi$. We periodically extend each $\mathcal{TI}$ curve to $\mathbb{R}$, showing the $\mathcal{TI}$ curves on $[0, 2\pi)$ in the red boxes. $\overline{R}_\varphi$ translates $\mathcal{TI}$ by φ (middle row) while $\overline{\kappa}_\varphi$ reflects $\mathcal{TI}$ across the axis $\theta = \varphi$ (bottom row).

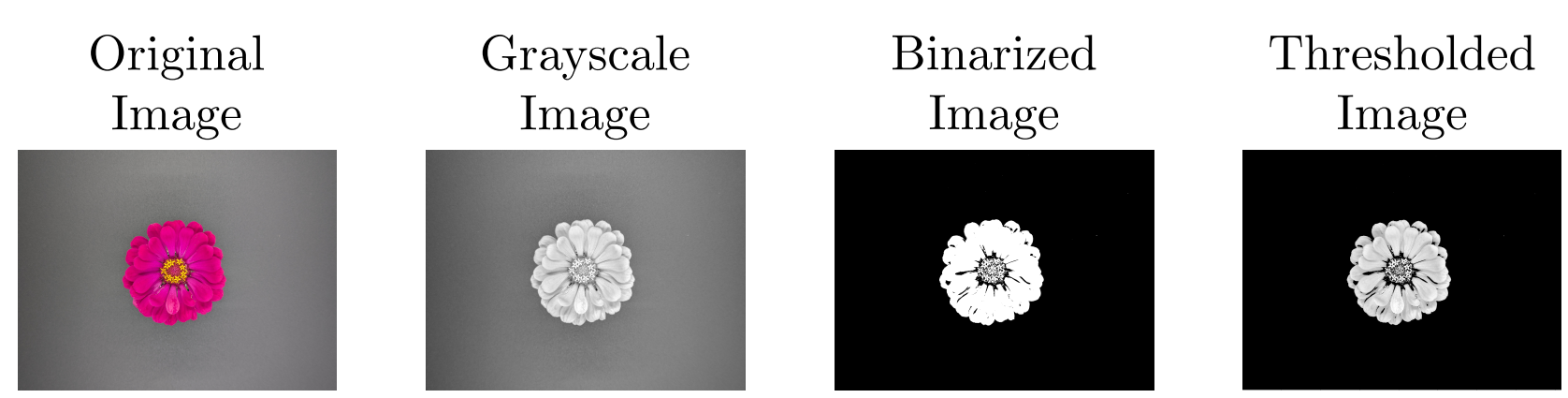

Figure 2: Here we show the difference between binarizing and thresholding as described in Section 7.3.

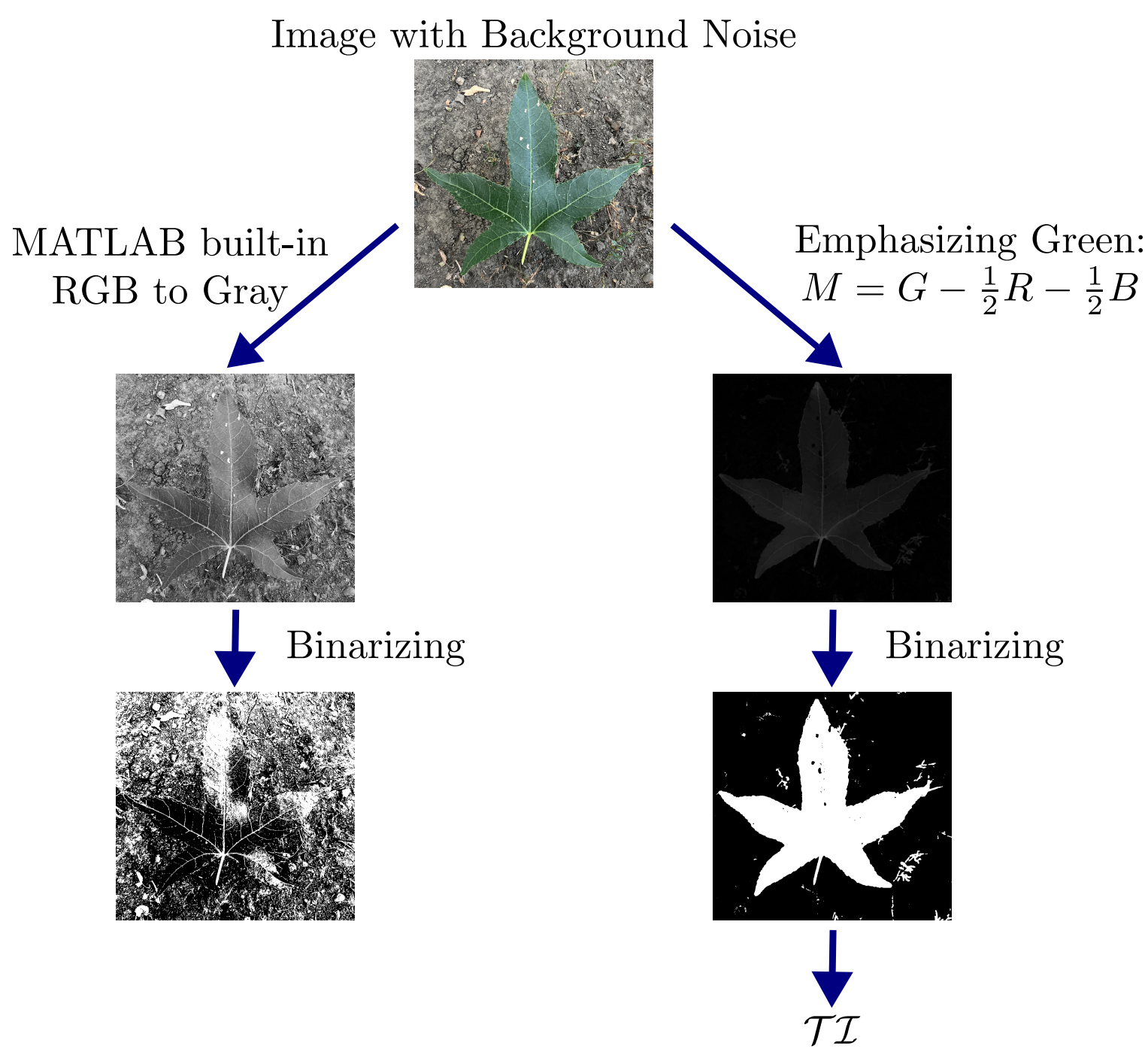

Figure 3: The built in RGB to Gray conversion in MATLAB is not sufficient to separate the object of interest from the background (left). Using a weighted sum that emphasizes green, we can separate the object from the background (right).

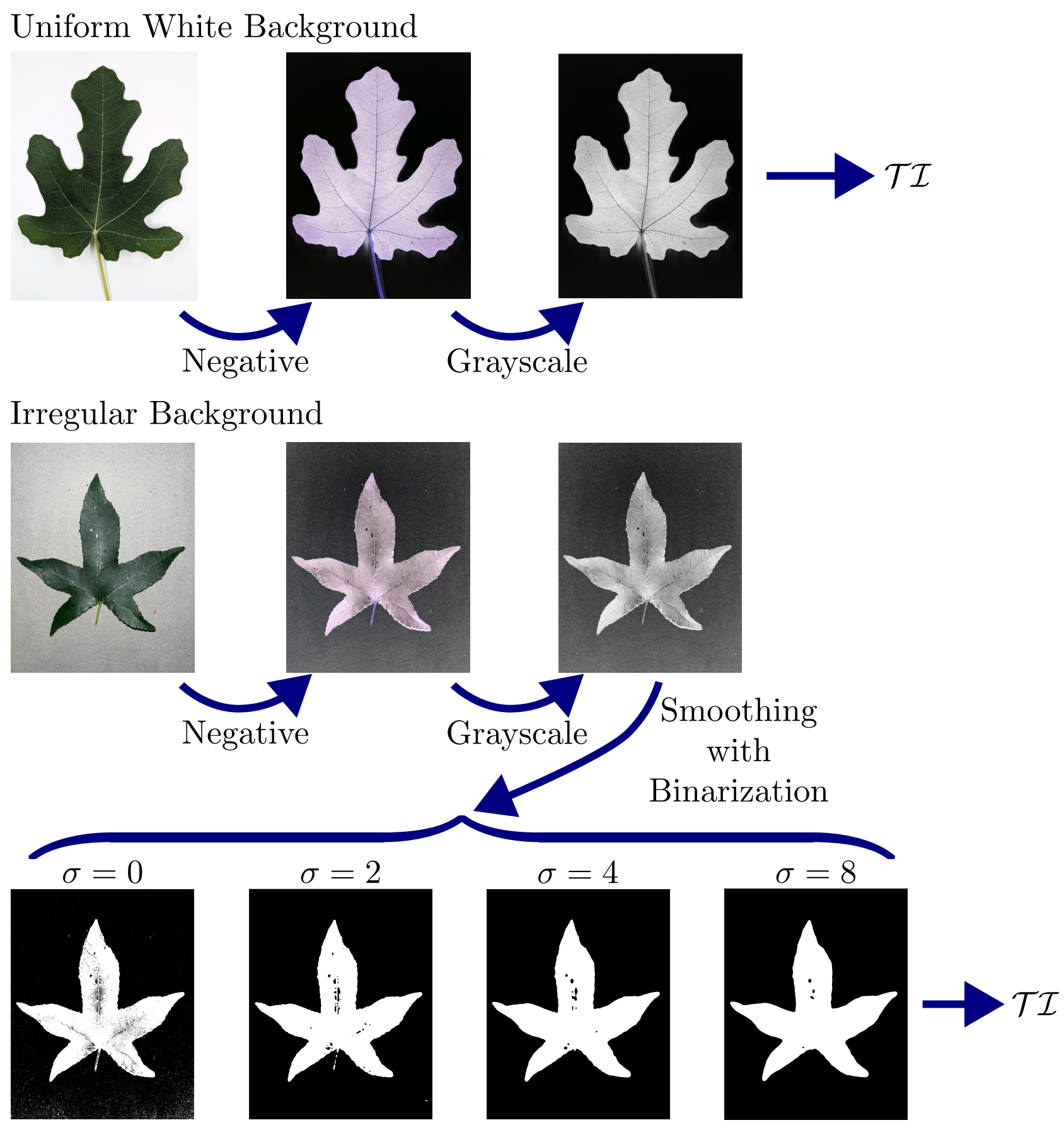

Figure 4: This figure illustrates several additional preprocessing procedures. When the image background is uniformly white (top row), we suggest taking the negative and the grayscale before computing $\mathcal{TI}$. If the background is irregular (middle row), smoothing and binarizing help isolate the object. The bottom row shows how varying the standard deviation σ in Gaussian smoothing influences the binarized image.
